# Inductively tuned modified split ring resonator based quad band epsilon negative (ENG) with near zero index (NZI) metamaterial for multiband antenna performance enhancement

**DOI:** 10.1038/s41598-021-91432-8

**Published:** 2021-06-07

**Authors:** Md Moniruzzaman, Mohammad Tariqul Islam, Norbahiah Misran, Md Samsuzzaman, Touhidul Alam, Muhammad E. H. Chowdhury

**Affiliations:** 1 Department of Electrical, Electronic and Systems Engineering, Faculty of Engineering and Built Environment, Universiti Kebangsaan Malaysia, Bangi, Malaysia; 2 Department of Computer and Communication Engineering, Faculty of Computer Science and Engineering, Patuakhali Science and Technology University, Dhaka, Bangladesh; 3 Pusat Sains Ankasa (ANGKASA), Institut Perubahan Iklim, Universiti Kebangsaan Malaysia, 43600 UKM Bangi, Selangor Malaysia; 4 Department of Electrical Engineering, Qatar University, 2713 Doha, Qatar

**Keywords:** Electrical and electronic engineering, Metamaterials

## Abstract

An inductively tuned modified split-ring resonator-based metamaterial (MTM) is presented in this article that provides multiple resonances covering S, C, X, and Ku-bands. The MTM is designed on an FR-4 substrate with a thickness of 1.5 mm and an electrical dimension of 0.063λ × 0.063λ where wavelength, λ is calculated at 2.38 GHz. The resonator part is a combination of three squared copper rings and one circular ring in which all the square rings are modified shaped, and the inner two rings are interconnected. The resonance frequency is tuned by adding inductive metal strips in parallel two vertical splits of the outer ring that causes a significant shift of resonances towards the lower frequencies and a highly effective medium ratio (EMR) of 15.75. Numerical simulation software CST microwave studio is used for the simulation and performance analysis of the proposed unit cell. The MTM unit cell exhibits six resonances of transmission coefficient (S_21_) at 2.38, 4.24, 5.98, 9.55, 12.1, and 14.34 GHz covering S, C, X, and Ku-bands with epsilon negative (ENG), near-zero permeability, and near-zero refractive index (NZI). The simulated result is validated by experiment with good agreement between them. The performance of the array of the unit cells is also investigated in both simulation and measurement. The equivalent circuit modeling has been accomplished using Advanced Design Software (ADS) that shows a similar S_21_ response compared to CST simulation. Noteworthy to mention that with the copper backplane, the same unit cell provides multiband absorption properties with four major absorption peaks of 99.6%, 95.7%, 99.9%, 92.7% with quality factors(Q-factor) of 28.4, 34.4, 23, and 32 at 3.98, 5.5, 11.73 and 13.47 GHz, respectively which can be applied for sensing and detecting purposes. The application of an array of the unit cells is investigated using it as a superstrate of an antenna that provides a 73% (average) increase of antenna gain. Due to its simple design, compact dimension with high EMR, ENG property with near-zero permeability, this multiband NZI metamaterial can be used for microwave applications, especially for multiband antenna gain enhancement.

## Introduction

Metamaterial, as an artificial media, shows some exotic properties acquired from geometry rather than composition. The effective macroscopic properties are achieved by inclusion or adding in homogeneities named “meta-atoms.” The properties include artificial plasmas, double negative, permeability negative, negative index media, to name a few. These characteristics find their applications in many areas, such as enhancing the performance of antenna^1^, reduction of specific absorption rate(SAR)^2^, filter design^3^, absorber^4^, sensing and detection^5^, energy harvesting^6^, super lensing^7^ and so on. The applications are also extended in large fields such as acoustic, optics, microwave, mechanical, electronics, chemical, biological^8–11^.

In the recent works, a split-ring resonator (SRR) based material is introduced by Meng et al. that is operable in THz frequency range and can be used to sensing dielectric. The mechanism for sensing is followed by etching trenches into the split gap, and thus sensitivity is observed by shifting the resonance frequency^12^. A greek key-shaped resonator-based multiband metamaterial has been presented by Zarghooni et al. that shows multiband resonances within 2–5 GHz^13^. With its 10 × 10 mm^2^ physical dimension, some unique features can be expressed as it offers a near-zero refractive index in the band of frequencies. On the other hand, an SRR based metamaterial has been employed in^14^ that has the potentiality to reduce the impedance of beam coupling in an accelerator. The SRR functions as a mode damper in the accelerating system that can be an alternative to other devices for impedance mitigation. The effect of electromagnetic coupling has been investigated by Wang et al. where U-shaped SRRs are fabricated on polyimide substrate to form toroidal dipole metamaterials applicable in terahertz devices^15^. A periodical metamaterial is waved in fabric material using ferromagnetic microwires to create a meta-composite that can be used for health monitoring of structure with X band electromagnetic response of X-band^16^. A metamaterial microwave absorber is presented in^17^ that is constructed with asymmetric sectional resonators of different sizes. This metamaterial absorber exhibits broadband near-unity absorption characteristics within 7–9 GHz. Cheng et al. demonstrated an anisotropic metamaterial composed of a bi-layered disk-split-ring array and sandwiched sub-wavelength metal grating. This metamaterial array acts as a polarization converter with more than 99% efficiency at 4.5 GHz and 7.9 GHz^18^. A patch antenna is presented in^19^, where metasurface is used as the focusing elements. Highly efficient polarization reflection of this metasurface helps to energy gathering that increases the gain. An array of metamaterial unit cells are used with an antenna to increase the bandwidth^20^. Moreover, several metamaterial structures have been described in the literature^21–23^ with effective parameter analysis, and these metamaterials can be used with microwave devices. In^24^, the metamaterial structure has been designed targeting liquid sensing applications. The frequency band obtained within 8–12 GHz can be used to detect different types of liquid chemicals by considering the effect on bandwidth shifting within this band for various chemicals. A complementary split-ring resonator-based metamaterial has been presented by Sharples et al., which acts as a Cherenkov detector in acceleration science^25^. An SRR based metamaterial sensor has been presented in^26^ that can be used to measure liquid permittivity. An oval-shaped metamaterial is introduced by MT Islam et al. that shows its resonance in S-band and can be used for glucose sensing^27^. A polyimide-based flexible metamaterial has been reported in^28^, where ferrite is incorporated to obtain high inductance and capacitance in SRR. This metamaterial demonstrates two resonances at 2 and 2.5 GHz, which is the outcome of the high permeability resulted from ferrite materials. A broadband metamaterial has been demonstrated by Zhang et al. that provides optical transparency along with absorption in the microwave region^29^. Recently a cross-coupled SRR based metamaterial has been reported in^30^ that provides three resonances at covering C, X and Ku-bands with epsilon negative properties and suitable for the applications in satellite and radar communication devices. A metamaterial is purposely designed by T. Shabbir et al. with the characteristics of negative permittivity and near-zero permeability and utilize to enhance the performance of UWB-MIMO antenna^31^. A dual-band metamaterial is reported in^32^, which is acted as a dual-band absorber. This MTM absorber provides near-unity absorption peaks at 9.45 and 9.8 GHz, respectively assisted with plasmon and Mie resonances. A tunable metamaterial is presented in^33^ where the tunable unit is integrated in background metallic layer. This MTM absorber shows modulated frequency depending upon the temperature, thus have the potentiality for temperature sensing and thermal emitters. A quad band MTM is presented in^34^ that acts as an microwave absorber with good selectivity, whereas in^35^ an epsilon negative metamaterial with high effective medium ratio is described for wireless communications in microwave region.

A new metamaterial is presented in this article consisting of four split rings providing six resonances covering S, C, X, and Ku-bands whose resonance is tuned by inductive metal strips connected in parallel with two splits in the outer ring. A novelty of this metamaterial is that the design is simple, and it provides ENG properties with near-zero permeability and refractive index. Moreover, it offers a high EMR of 15.63, indicating the compactness of this design. Furthermore, it provides multiband absorption properties with a full copper backplane exhibiting major absorption peaks of 99.6%, 95.7%, 99.9%, and 92.7% with Q-factors of 28.4, 34.4, 23, and 32 at 3.98, 5.5, and 11.73, 13.47 GHz, respectively. The rest of the paper is organized as follows. In section two, the design and simulation method is presented with the impact of different design steps, whereas theory on metamaterial property extraction is described in section three. In section four, the surface current, electric, and magnetic fields are analyzed, and section five is dedicated for evaluating equivalent circuit of the proposed MTM unit cell. A parametric study is presented in section six, and in the next section, results with discussion are made. In the subsequent sections, the application of this MTM in the antenna is discussed, followed by the conclusion in the final section.

## Design and simulation of the proposed MTM unit cell

**Figure 1:**
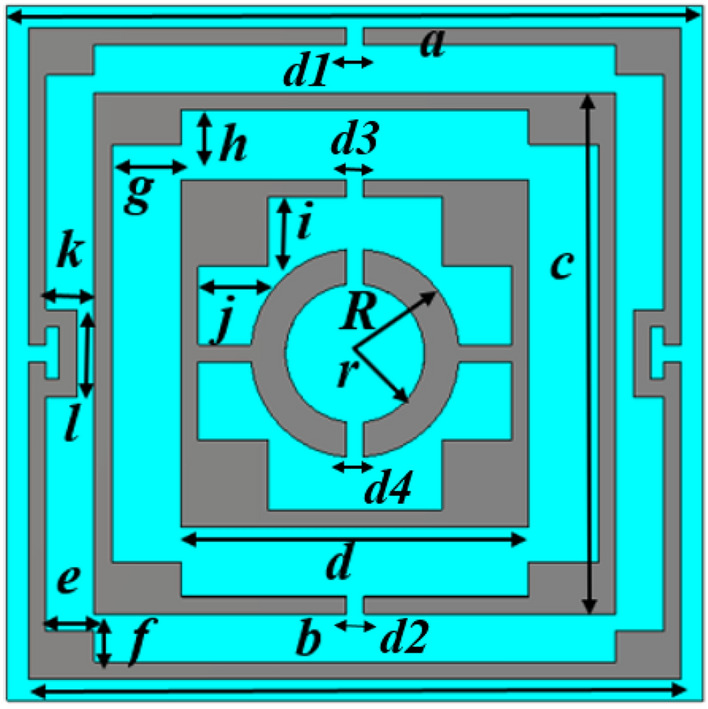
Layout of the proposed unit cell.

**Figure 2:**
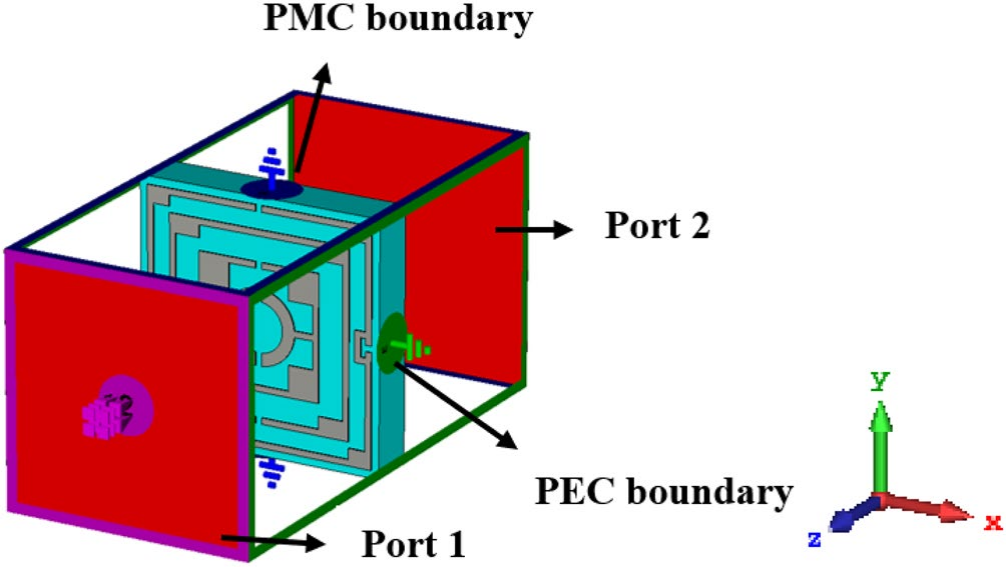
Simulation setup of the unit cell.

**Figure 3:**
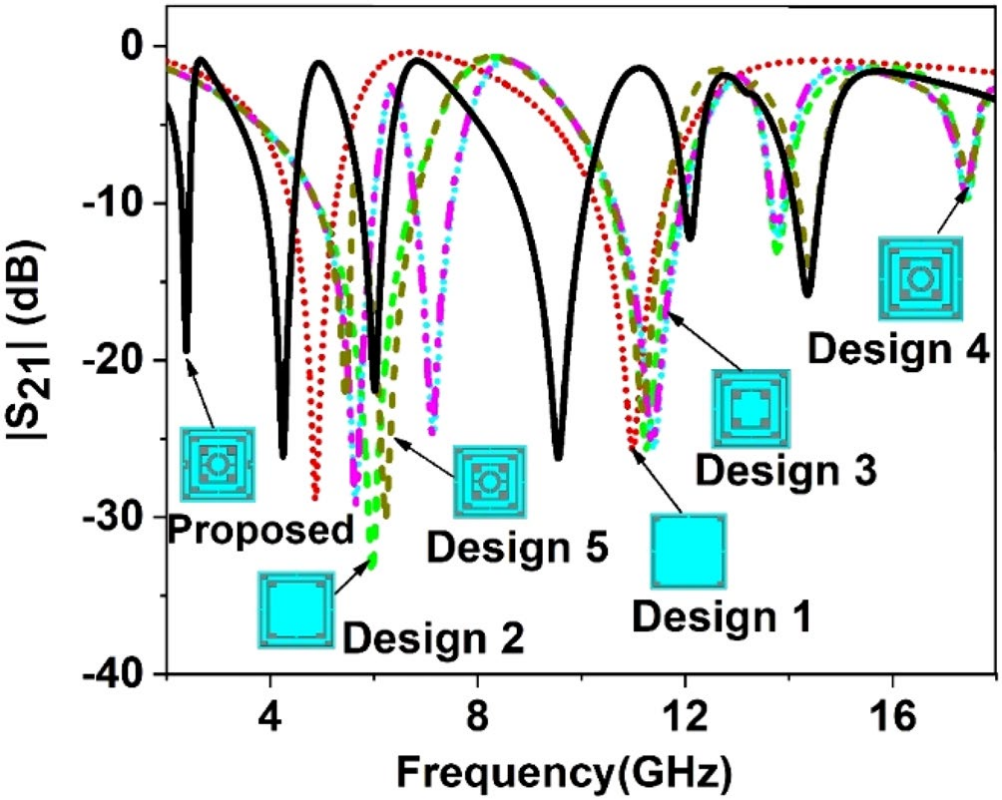
Change of |S_21_| for various design steps.

The effective response of the metamaterial can be obtained if the unit cell dimension is small enough compared to the wavelength at the operating frequency^36^. It is intended to design a metamaterial that will provide multiband resonances, so the dimension is selected at the lowest probable operating frequency. In this design, the target bands are S, C, X, and Ku bands, where the lowest frequency band is S-band that extends from 2–4 GHz. So, the initial dimension of the unit cell is estimated at 2 GHz (the wavelength, λ_L,_ at this frequency is 150 mm). A choice of unit cell length, L of 8 mm indicates that L = λ_L_/18.75 that is small enough to fulfill the sub-wavelength criteria of the metamaterial. The unit cell structure is chosen from the conventional split ring resonator in a modified form where the innermost two rings are interconnected. The split gaps and interconnecting points are so selected that the unit cell is axis-symmetric. Moreover, shunt inducting cooper strips are included in parallel to the splits gaps of the outer ring, which helps to tune the resonance frequencies. The resonance frequency can be modified by changing the area of these inducting metal strips; thus, the inductively tuned property is obtained. Now, the design of the proposed metamaterial unit cell is initiated on an FR-4 substrate with a length and width of 8 mm, each having thickness of 1.5 mm. The substrate exhibits its properties of permittivity 4.4 with a loss tangent of 0.02. At both sides of the substrate, there is a copper layer of 0.035 mm thickness. For metamaterial design, one side of it is made copper-free, and on another side, it contains the resonator. The resonator comprises four split rings within those three are square-shaped, and another one is circular. All the square-shaped rings have an extended rectangle at each corner, increasing the surface area of the rings. The outer ring includes three splits within which two shunt copper paths are used parallel to the splits to modify the resonance frequencies. All the square rings are of equal width. A metal stub interconnects the third square ring and innermost circular ring. The design is mirror-symmetric to the vertical axis, and all the split gaps are of equal length. Figure 1 shows the proposed unit cell's design layout, and the various dimensions of different segments and gaps are listed in Table 1. The unit cell simulation is performed in frequency domain solver using numerical simulation software CST microwave studio suite, 2019. The simulation arrangement is presented in Fig. 2, where the incident electromagnetic wave is applied along the z-axis, whereas the x-axis is utilized for electric field boundary and the y-axis for magnetic field boundary. The electric boundary operates as a perfect electric conductor with the tangent component of electric fields and normal components of the magnetic fields having zero values. Similarly, magnetic boundaries are perfect, with the tangential components of magnetic fields and normal components of electric fields having zero values. A plane wave of transverse electromagnetic (TEM) mode is exposed on the resonator to analyze the scattering behavior of the MTM. The unit cell design undergoes different design evolution steps as presented in Fig. 3 to obtain the desired transmission coefficient (S_21_). Design 1, illustrated in Fig. 3, contains only one ring with three split gaps. This ring with multiple gaps forms multiple capacitive elements with inductors that exhibit dual resonances at around 5 and 11 GHz. In design 2 of the same Figure, another ring is added inside the first ring. The inclusion of the second ring causes shift of earlier two resonances at 5.9 GHz and 11.28 GHz. The mutual coupling effect between these two rings is the reason for this shifting. The inclusion of the second ring also causes another additional resonance at 13.8 GHz. In the subsequent step, at design 3 in Fig. 3, a third ring is added to shift the lower resonance frequency towards 5.7 GHz and the next resonance toward 11.4 GHz.

**Table 1:** Various dimensions of the different segments and split gap of the proposed unit cell.

Parameter	Dimension (mm)	Parameter	Dimension (mm)	Parameter	Dimension (mm)	Parameter	Dimension (mm)
*a*	8.0	*b*	7.8	*c*	6.0	*d*	4.0
*e*	0.55	*f*	0.35	*g*	0.8	*h*	0.4
*i*	0.8	*j*	0.8	*k*	0.35	*l*	1.0
*d1, d2*	0.2, 0.2	*d3, d4*	0.2, 0.2	*r*	0.8	*R*	1.2

The resonance at 13.8 GHz is unaffected in this design step, and the inclusion of the third ring adds another resonance around 7 GHz. Thus, every subsequent addition of the ring contributes to a new resonance. Next, a circular split ring is added inside the third ring, as shown in design 4 of Fig. 3. This ring shows an impact of shifting all resonances a little bit with the addition of a new resonance around 17.5 GHz with an amplitude of − 9.3 dB. In the next step, the third square ring and innermost circular ring are interconnected at both sides by using two metal strips as shown in design 5 in Fig. 3. Joining the two rings with two metal strips alters the resonance frequencies. Finally, in the proposed design, two conducting paths are created in parallel to the two vertical splits of the outmost ring that causes a drastic shift of all the resonances towards the lower values, as shown in Fig. 3. The outcomes of different design steps are summarized in Table 2.

**Table 2:** Summary of S_21_ properties for various design steps of the proposed metamaterial unit cell.

Steps	Resonance frequency (GHz)	Amplitude(dB)	Covering Band
Design 1	4.9, 11.15	− 28.9, − 26.2	C, X bands
Design 2	5.95, 11.3, 13.8, 17.5	− 33.5, − 25.3, − 13, − 9.7	C, X, Ku bands
Design 3	5.65, 7.2, 11.4, 13.8, 17.4	− 29, − 24.7, − 25.5, − 12, − 9.4	C, X, Ku bands
Design 4	5.65, 7.14, 11.4, 13.8, 17.4	− 29, − 24.4, − 25.4, − 11.7, − 9.4	C, X, Ku bands
Design 5	5.5, 6.2, 11.2, 14.4, 17.5	− 22.5, − 30.1, − 22.9, − 13.9, − 8.4	C, X, Ku bands
Proposed	2.38, 4.24, 5.98, 9.55, 12.1, 14.34	− 19.7, − 26.0, − 21.8, − 26.1, − 12.0, − 15.7	S, C, X, Ku bands

## Metamaterial property extraction method

The properties of any material are frequency-dependent, for these reasons different models have been designed to describe the frequency response of the material. In most of the material, the electric field intensity is high compared to the magnetic field due to the wave impedance; thus, the focus is given to the electron motion in the atoms, which causes the change of dipole moment. Thus, the model is introduced based on the electrical susceptibility of that medium. Contrary to this, there are many media in which the magnetic field is more dominant compared to the electric field. In that case, it is described the magnetic field response in terms of magnetic susceptibility in dual of the electric field since magnetic dipole is created by the moments of the electric current loop. The most popular model for material characterization is the Lorentz model in which motion of the electron in a material is described in terms of the damped, excited harmonic oscillator having polarization field that can be expressed in relation to the electric field as^37^:

1 $$\frac{{d^{2} }}{{dt^{2} }}P_{i} + {\Gamma }_{L} \frac{d}{dt}P_{i} + \omega_{0}^{2} P_{i} = \varepsilon_{0} \chi_{L} E_{i}$$

In which the first term of the left side indicates acceleration of charges, second one damping mechanism with damping coefficient, $${\Gamma }_{L}$$ and the third one for restoring force with a characteristic frequency, $$f_{0} = {\raise0.7ex\hbox{${\omega_{0} }$} \!\mathord{\left/ {\vphantom {{\omega_{0} } {2\pi }}}\right.\kern-\nulldelimiterspace} \!\lower0.7ex\hbox{${2\pi }$}}$$. On the right side of Eq. (1), driving terms is included where $$\chi_{L}$$ is the coupling coefficient. By performing the necessary mathematical computation, the solution of the polarization field $$P_{i}$$ can be obtained in terms of the electric field, $$E_{i}$$ and from this solution, electric susceptibility can be obtained as^37^:

2 $$\chi_{e, Lorentz} \left( \omega \right) = \frac{{P_{i} \left( \omega \right)}}{{\varepsilon_{0} E_{i} \left( \omega \right)}} = \frac{{\chi_{L} }}{{ - \omega^{2} + j{\Gamma }_{L} \omega + \omega_{0}^{2} }}$$

Now by using Eq. (2), permittivity can be defined as:

3 $$\varepsilon_{Lorentz} \left( \omega \right) = \varepsilon_{0} \left[ {1 + \chi_{e, Lorentz} \left( \omega \right)} \right]$$

Neglecting the restoring force in Eq. (1), Drude model can be formed from which expression of electric susceptibility will be^37^:

4 $$\chi_{e, Drude} \left( \omega \right) = \frac{{\chi_{D} }}{{j{\Gamma }_{D} \omega + \omega_{0}^{2} }}$$

in which the coupling coefficient is generally presented by the plasma frequency, $\chi_{D} = \omega_{p}^{2}$. From these two models, negative permittivity can be obtained considering that the coupling coefficient is positive. The resonant nature of the Lorentz model causes the real part of susceptibility to be negative, which in turn makes permittivity negative in a narrow band above the resonance frequency. Contrary to this, Wide spectral negative permittivity can be obtained in the Drude model for $\omega < \sqrt {\omega_{p}^{2} - {\Gamma }_{D}^{2} } .$

Similarly, magnetic counterpart can be calculated by considering relative magnetic terms such as magnetic polarization, magnetic field instead of electrical ones in Eq. (1), magnetic susceptibility, $\chi_{m}$ can be calculated, and permeability can be represented as:

5 $$\mu_{ } \left( \omega \right) = \mu_{0} \left[ {1 + \chi_{m} \left( \omega \right)} \right]$$

The Drude-Lorentz variables regarding the material properties are parameterized by using CST microwave studio. Considering S parameters is the optimum goal, optimization is carried out by iterative simulation in CST for identifying the parameters that incorporate with the values obtained Drude-Lorentz model and replicate best with the behavior of the unit cell^38^. The metamaterial properties such as permittivity, permeability, refractive index, impedance can be obtained by using numerous methods such as robust retrieval^39^ method, Nicolson-Ross-Wier (NRW)^40,41^ method by using transmission coefficient, S_21_ and reflection coefficient, S_11_ obtained from the simulation in CST. In this article, the most used method, i.e., NRW is used for this purpose. To obtain the expression of effective parameters to terms V_1_ and V_2_ are determined by addition and subtraction of S_21_ and S_11_|, respectively.

6 $$V_{1} = |S_{11|} + |S_{21} |$$

7 $$V_{2} = \left| {S_{21} \left| - \right|S_{11} } \right|$$

$${\text{if}}{\kern 1pt} X = \frac{{1 - V_{1} V_{2} }}{{V_{1} - V_{2} }}$$

Then reflection coefficient of the incident wave at the interface,

8 $${\Gamma } = {\text{X}} \pm \sqrt {X^{2} - 1}$$

And the transmission coefficient,

9 $$X = \frac{{V_{1} - {\Gamma }}}{{1 - V_{1} {\Gamma }}}$$

The expression for refractive index, n, and impedance, z can be derived from these equations. The relative permittivity, ε_r_, and relative permeability, µ_r_ can also be extracted and represented in the form of Eqs. (10) and (11) ^42^.

10 $$\varepsilon_{r} \sim\frac{2}{{jk_{0} d}} \times \frac{{\left( {1 - V_{1} } \right)}}{{\left( {1 + V_{1} } \right)}}$$

11 $$\mu_{r} \sim\frac{2}{{jk_{0} d}} \times \frac{{\left( {1 - V_{2} } \right)}}{{\left( {1 + V_{2} } \right)}}$$

where $k_{0} = \frac{2\pi f}{c}$, c is the velocity of light, and d is the thickness of the substrate.

The expression for the refractive index, n_r_ is represented in Eq. (12).

12 $$n_{r} = \sqrt {\varepsilon_{r} \mu_{r} }$$

Using MATLAB code based on the Eqs. (10)–(12) in corporation of S parameters obtained from CST microwave studio can be used to determine the permittivity, permeability, and refractive index of the proposed metamaterial unit cell to further analysis and utilization of these properties.

## Surface current, electric and magnetic field analysis of the designed MTM unit cell

The resonance phenomena in a metamaterial can be explained considering the interrelation between the electric field, current, and magnetic field in a metamaterial using Maxwell equations. If electric charge differs with time, it may act as a source of the magnetic field. In a reverse way, this varying magnetic field provides a time-varying electric field as an outcome. Thus, electric current, fields of electricity, and magnetism are associated with each other, and the distribution of these currents and fields in the material impacts the resonance phenomena.

**Figure 4:**
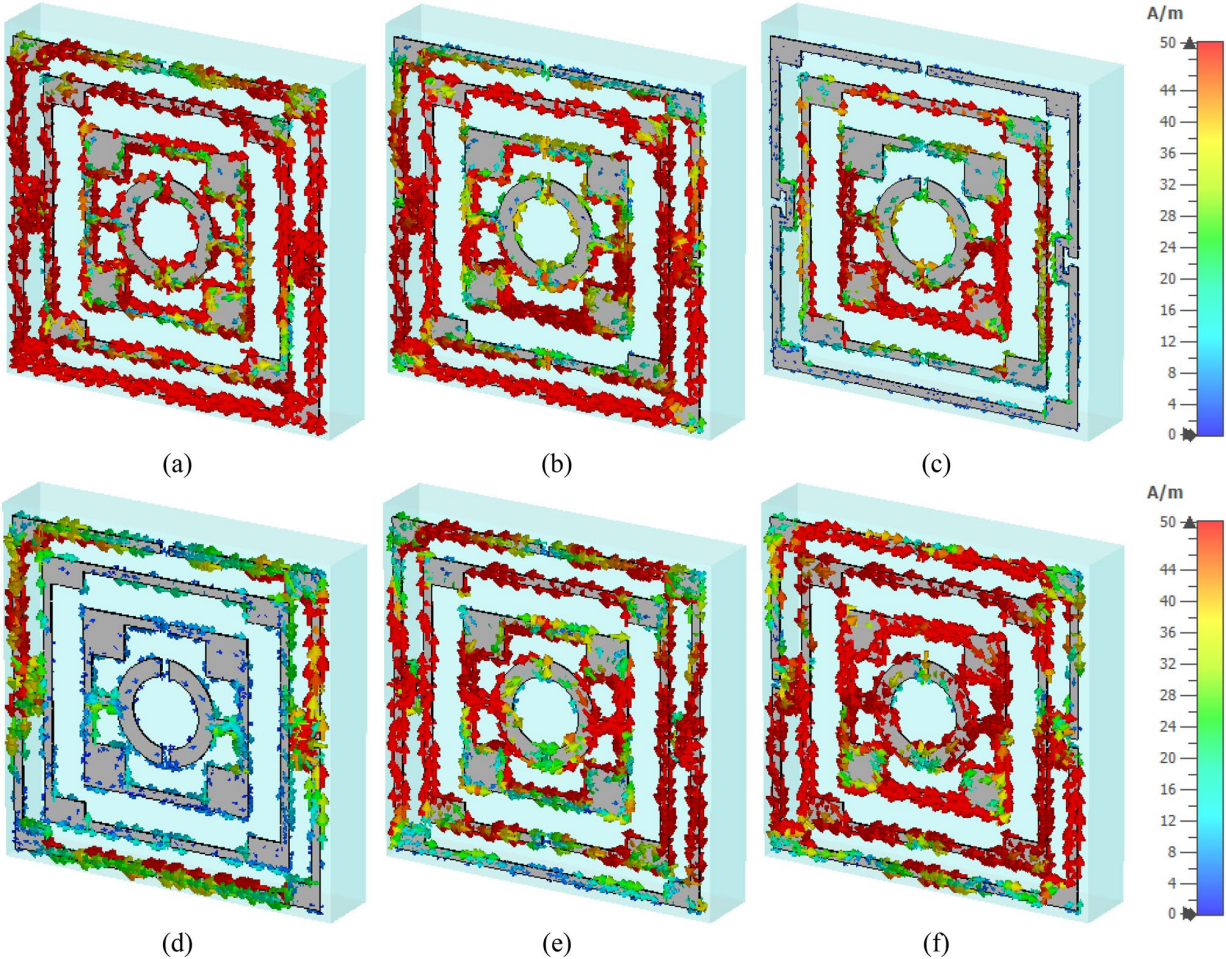
Surface current distribution of the unit cell (**a**) 2.38 GHz, (**b**) 4.24 GHz, (**c**) 5.98 GHz, (**d**) 9.55 GHz, (**e**) 12.1 GHz, and (**f**) 14.34 GHz.

**Figure 5:**
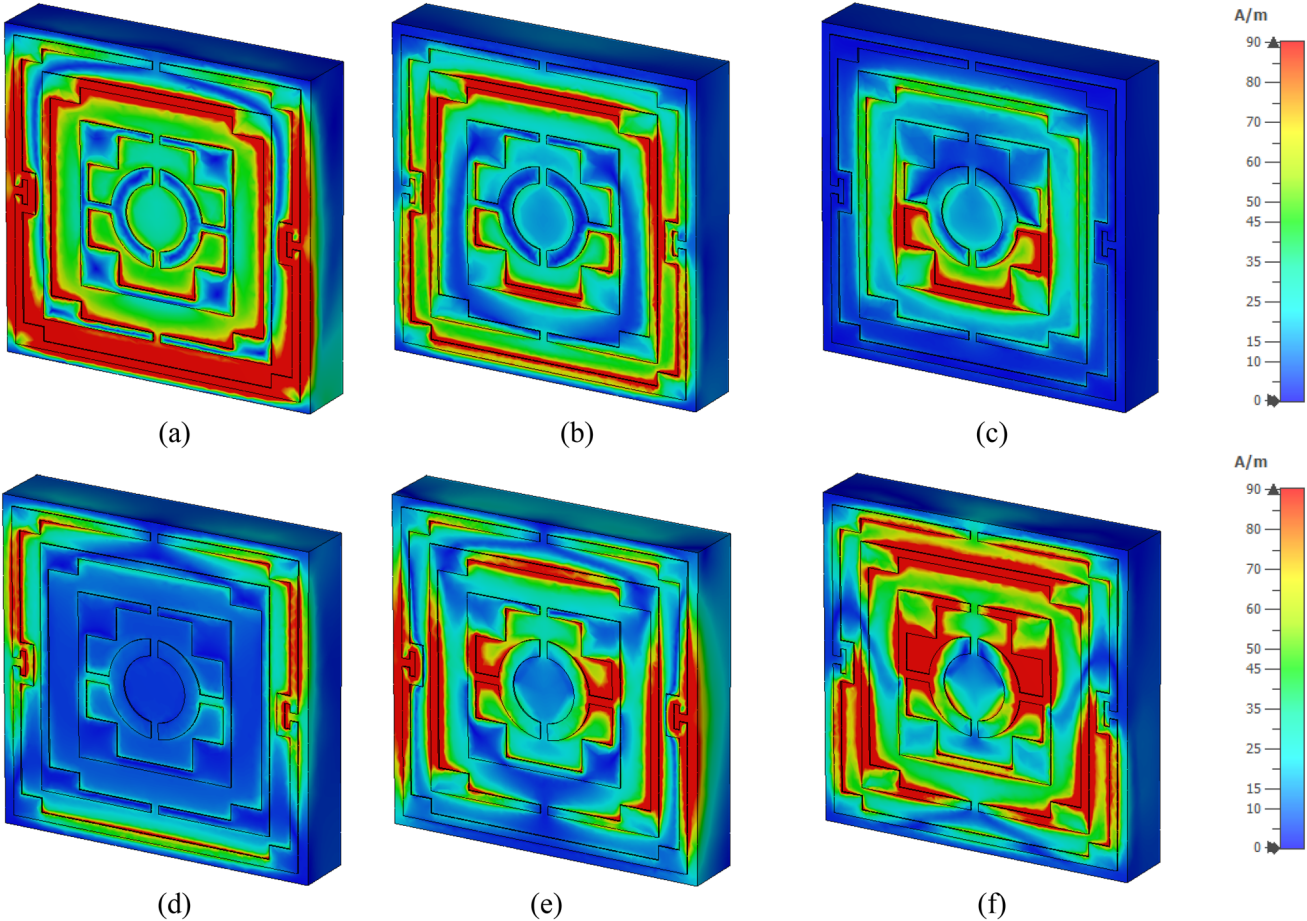
H field distribution of the unit cell (**a**) 2.38 GHz, (**b**) 4.24 GHz, (**c**) 5.98 GHz, (**d** ) 9.55 GHz, (**e**) 12.1 GHz, and (**f**) 14.34 GHz.

**Figure 6:**
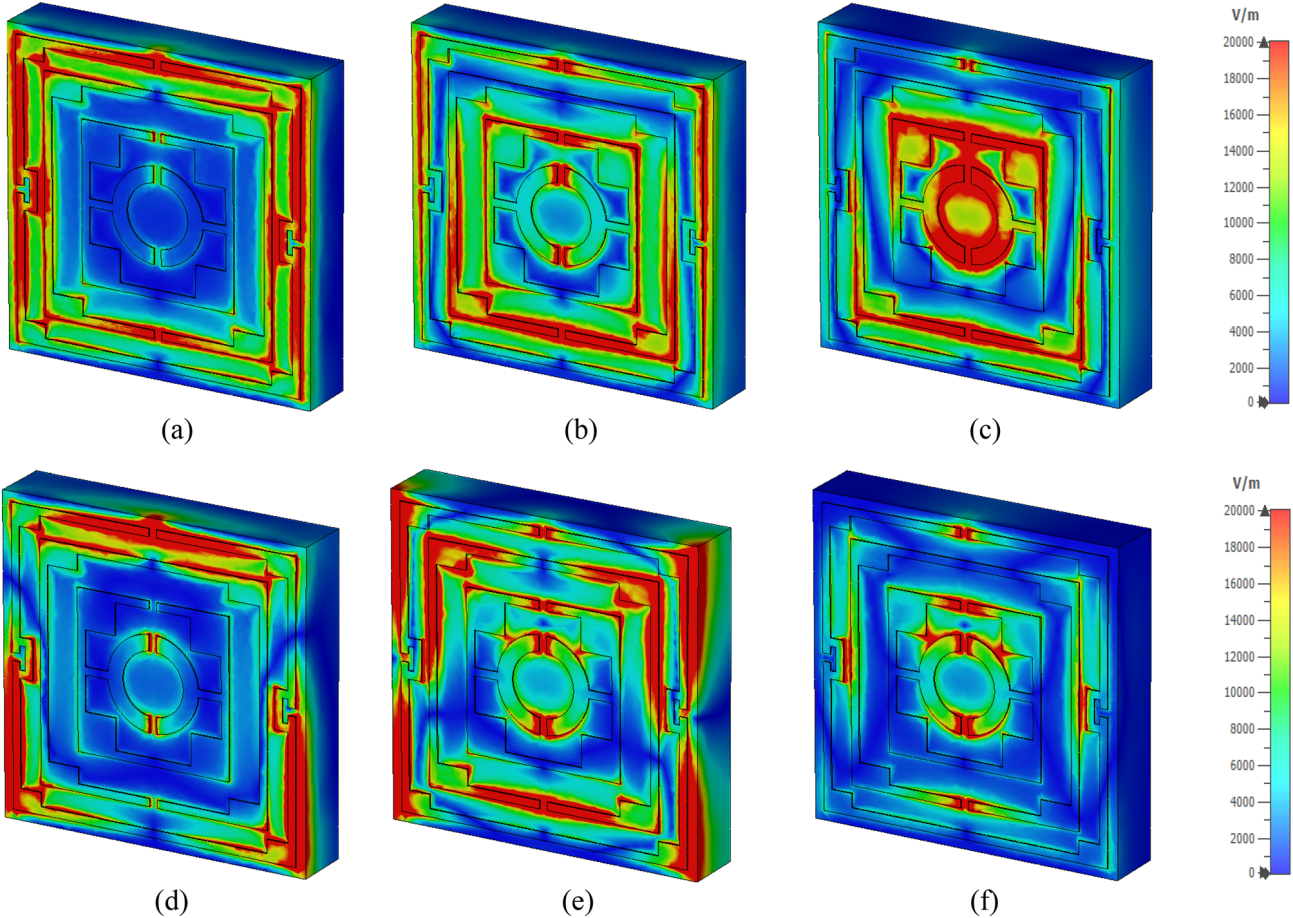
E field distribution of the proposed unit cell: (**a**) 2.38 GHz, (**b**) 4.24 GHz, (**c**) 5.98 GHz, (**d**) 9.55 GHz, (**e**) 12.1 GHz, and (**f**) 14.34 GHz.

The surface current distributions for different resonance frequencies are shown in Fig. 4. At 2.38 GHz, the surface current is high in the outer ring, whereas the second ring contributes a significant amount of current. The currents in these two rings are parallel to each other, which tries to amplify the magnetic field induced by them. Thus, a strong mutually coupled magnetic field is observed around these two rings, as shown in Fig. 5a. Whereas the currents through the inner two rings are antiparallel, thus it neutralizes their magnetic field effect. So, the magnetic field around the two inner rings is very low. An investigation in the electric field (shown in Fig. 6a) reveals that a strong electric field exists in the vicinity of the two outer rings. Thus, electric and magnetic resonances occur due to the high electromagnetic field that causes resonances in scattering parameters along with permittivity and permeability in that frequency. As shown in Fig. 4b, the current decreases in the first ring, and an antiparallel current is noticed in the second ring. Thus, the magnetic effect between the first and second ring decreases compared to the previous frequency, as shown in Fig. 5b. Two current loops are formed between the third and innermost ring along with the coupling between the two rings. In both loops, current flows in the clockwise direction with a comparatively higher current in the lower loop. Thus, they both contribute to the same directional magnetic field with less contribution by the upper loop. It is also observed that the loop current is in the same direction at the second resonator. As the frequency increases, the current in the first and second ring decreases, and at 5.98 GHz, the current through the first ring becomes nearly zero, whereas the current distribution in the third ring increases significantly. Two coupling stubs of the third and innermost ring also contain a large density of the surface current, as depicted in Fig. 4c. A drastic shift in current distribution is noticed in Fig. 4d, at 9.55 GHz, where the outer ring contains a moderate amount of current, but current density is very low in all the inner rings. A substantial effect is observed at the resonance frequency, 12.1 GHz, at which all the rings contain a significant amount of current, as presented in Fig. 4e. A dominant current in two vertical sides of the outer ring triggers the resonance at this frequency. Finally, as shown in Fig. 4f, current concentration increases in the inner rings, and the overall distribution of dense current in the second rind contributes to the resonance at 14.34 GHz.

Figure 5a–f exhibits magnetic field distribution for different resonance frequencies. The dependency of magnetic field on the current density is experienced by comparing these magnetic fields' distribution to the current density, as shown in Fig. 4. Figure 5a shows that a widespread magnetic field is experienced in the outer split ring due to the higher surface current density at 2.38 GHz. A strong magnetic field is observed in the second ring though this field is concentrated in narrow strips all over the ring. Subsequently, surrounding all the other rings magnetic field is low due to the lower current in these rings. At 4.24 GHz, the magnetic fields at the first and second ring decrease, and the lower half of the third ring exhibits an increasing pattern, as shown in Fig. 5b. At 5.98 GHz, a dominant magnetic field is noticed at the lower half of the third ring, as shown in Fig. 5c. The magnetic field at 9.55 GHz fades away in all inner rings, whereas a moderate field is observed at the outer ring (Fig. 5d). As the frequency increases, the magnetic field in all the rings increases gradually. At 12.1 GHz, a strong magnetic field is noticed in a narrow region on the vertical sides of the first and second ring (Fig. 5e). At the same time, at the coupling line of the third and innermost ring, the magnetic field is high. As presented in Fig. 5f, a widespread magnetic field is observed in different portions of the resonator, obeying the electromagnetic relation shown in Maxwell’s eqautions^36^. The electric field distribution is presented in Fig. 6a–f, and close observation of this Figure is expressed that strong electric fields are observed at the split gaps. These electric fields are due to the charge deposited near the split gaps for capacitive effects. Figure 6a shows that electric fields are low near the split gaps of the third and innermost rings. Due to low current flow in these two rings charge near the split gap is low causing lower strength of the electric field. It is also observed that electric field distribution is also closely related to the rate of change of the magnetic field, implying that where the rate of change in magnetic field distribution is high, the electric field is high. Thus, it satisfies the electric magnetic field relation presented by Maxwell’s equations^36^. Thus, the contribution of different portions of the resonator to electromagnetic characteristics triggers the resonance of S_21_ at different frequencies.

## Equivalent circuit modeling of the MTM unit cell

The SRR can be considered as a resonator that is coupled with the electromagnetic wave by electromagnetic induction. It may be modeled as an LC circuit that provides resonances at some specified frequencies determined by the inductance and capacitance offered by the SRR. The frequency(*f*) at which resonance occurs can be presented in the following form^43^:

13 $$f = \frac{1}{{2\pi \sqrt {L_{T} C_{T} } }}$$

where $L_{T}$ represents total inductance, which is a combination of self-inductance, $L_{S}$ by a resonator loop and mutual inductance, $L_{M}$ formed due to conducting arms of two adjacent SRRs. Therefore, the self-inductance can be expressed as^44^:

14 $$L_{S} = 0.2\mu_{0} \left( { - \frac{w}{2}sinh^{ - 1} 1 + \frac{w}{2}\sqrt 2 + \left( {l - \frac{w}{2}} \right)sinh^{ - 1} \left( {\frac{{l - \frac{w}{2}}}{\frac{w}{2}}} \right) - \sqrt {\left( {l - \frac{w}{2}} \right)^{2} + \left( \frac{w}{2} \right)^{2} } } \right)H$$

Similarly, mutual inductance can be represented as^44^:

15 $$L_{M} = 0.2.l_{ } \left( {ln\left( {\frac{l}{d} + \sqrt {1 + \frac{{l^{2} }}{{d^{2} }}} } \right) - \sqrt {1 + \frac{{d^{2} }}{{l^{2} }}} + \frac{d}{l}} \right)\mu H$$

where L is the side length, w is the width of the ring, and d is the center to center distance between adjacent sides of two SRRs. For calculating total capacitance, it is necessary to consider parallel plate capacitance formed due to the split gap and co-planar capacitance created between two adjacent SRR. Parallel plate capacitance due to the split gap in an SRR can be written as: $C_{ } = \varepsilon \left( \frac{A}{d} \right)$ where A is the area of the plate and d is the separation between the plate. The coplanar capacitance of unit length between two metal strips having a width, *p*, and distance between them, *q* can be represented by using the following equation^44^:

16 $$C_{cp} = \frac{{\left( {\varepsilon_{r} + 1} \right)\varepsilon_{0} }}{2}\frac{1}{\pi }\ln \left[ { 2\frac{1 + \sqrt k }{{1 - \sqrt k }}} \right]^{ }$$

where $k = \sqrt {\left( {1 - \left( {\frac{p}{p + 2q}} \right)^{2 } } \right)}$ is the geometrical factor.

**Figure 7:**
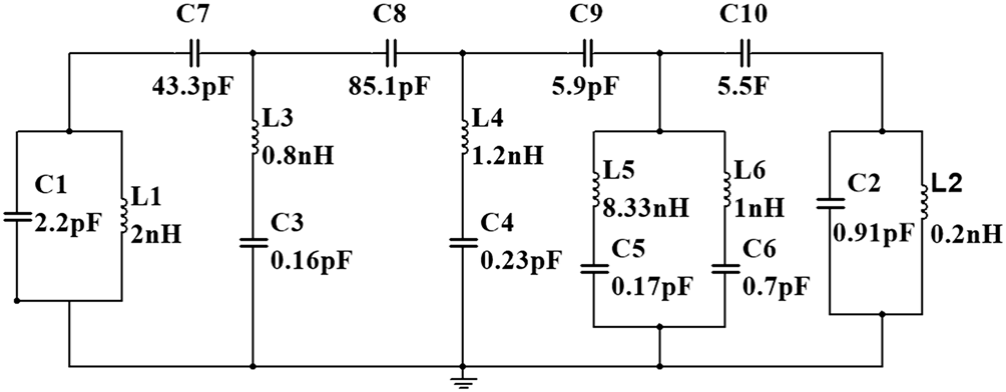
Equivalent circuit of the proposed MTM unit cell.

**Figure 8:**
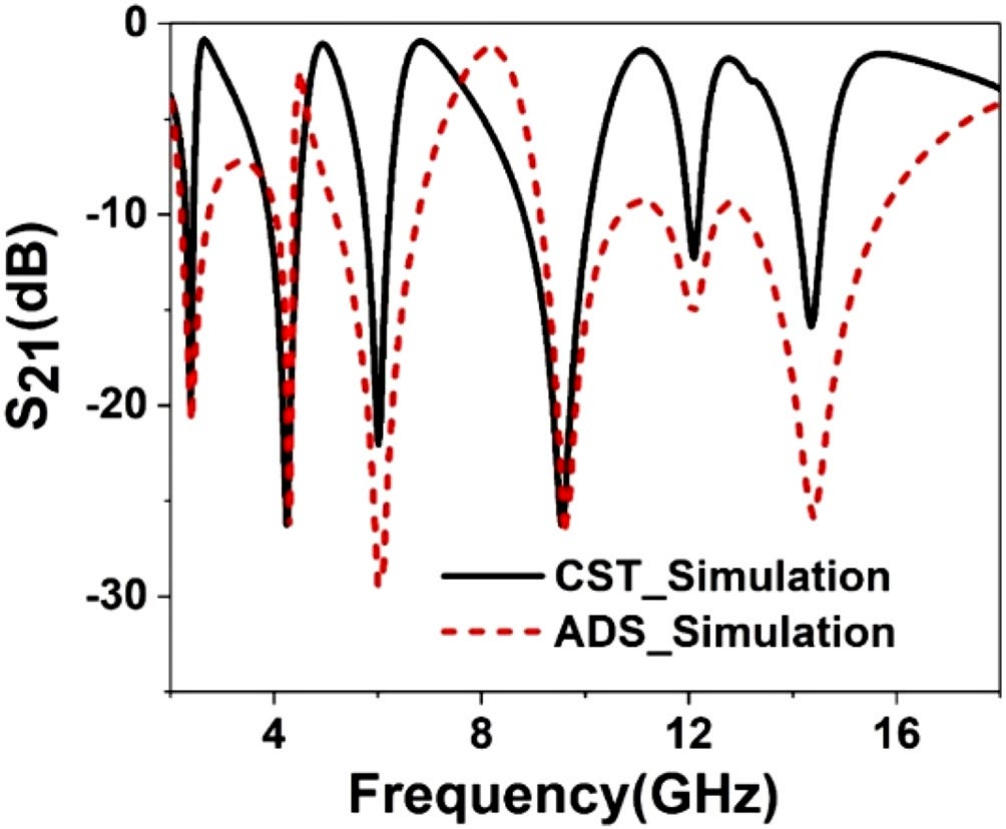
S_21_ comparison between ADS and CST Simulation.

The equivalent circuit of the proposed unit cell is presented in Fig. 7. The equivalent circuit is drawn based on the transmission line theorem where each metal part has the effect of inductance and split gap act as the capacitor. In this circuit, inductors *L1*, *L2,* and capacitors *C1*, *C2* are the equivalent circuit components of the outer ring of the unit cell. *L1*, *C1* pair acts as the parallel resonance circuit representing the equivalent circuit for the left half of this ring. These two components control the resonance frequency at 2.38 GHz, whereas *L2*, *C2* is the parallel resonance circuit for the right potion that controls the resonance at 12.1 GHz. *C7* and *C10* are the equivalent capacitance for the upper horizontal split gap of this ring. The impact of these two capacitances is that they control the shape of the waveform and magnitude at these two resonance frequencies. The inductor-capacitor pair *L3* and *C3* is the equivalent of the second ring of the unit cell, which triggers the resonance at 14.34 GHz. By controlling the values of these two components, resonance frequency and amplitude of S_21_ at this frequency can be controlled. The third ring can be presented by equivalent circuit components *L4*, *C4*, which are responsible for the resonance around 9.55 GHz. Finally, two splits of the innermost ring along with two metal strips are presented by two parallel LC circuits wherein each parallel branch corresponding circuit components are connected in series. Circuit components *L5*, *C5,* and *L6*, *C6* represent the equivalent components for this ring. L6 and *C6* play an important role in controlling the magnitude and resonance frequency around 5.98 GHz, whereas the resonance at 4.24 GHz can be tuned by the inductor, *L5,* and capacitor *C5*. C7 and *C8* are the co-planner capacitances between two adjacent ring segments. *C9* and C10 are associated with the parasitic capacitances among different copper segments. Though these capacitances are distributed in the patch for simplicity, it is shown as lumped components. These coplanar and parasitic components exhibit their impact on total magnitude. The above-discussed effect has been studied by simulating the circuit in Advanced Design Systems (ADS) software. The initial values of the most circuit components are obtained by using Eqs. (14) to (16). The component values obtained from these equations are *L1* = *L2* = 2.17nH, *L3* = 0.85nH, *L4* = 1.25nH *L6* = 1.4 nH, *C1* = *C2* = 2.45pF, *C3* = 0.26 pF, *C4* = 0.14 pF, *C5* = 0.15 pF, *C7* = 36.6 pF, *C8* = 30 pF. The equivalent circuit is drawn by using ADS considering these initial circuit component values. Then, final component values have been determined by tuning the component values in ADS so that the S_21_ of this circuit can be well-matched with the CST output. The magnitude of S_21_ can be adjusted by proper tuning of the series resistances associated with each inductor. But drawing simplicity, these values are ignored in the equivalent circuit, as shown in Fig. 7. The comparison of S_21_ between the outcome of ADS and CST is presented in Fig. 8. A close similarity between these two curves is observed from this Figure that validates the equivalent circuit as the representation of the proposed unit cell. A comparison between the circuit components values obtained from the Eqs. (14)–(16) and from the ADS circuit exhibits a mismatch between the component values. It is due to the fact that the proposed design is a modified form of a standard split ring resonator in which various inductive and capacitive values are distributive in nature, and inter-ring distance is not constant at every place. Thus, Eqs. (14)–(16) provide an approximation of the actual component values. On the other hand, in the ADS equivalent circuit presented in Fig. 7, circuit components are considered as lumped elements.

## Parametric study of the designed MTM unit cell

**Figure 9:**
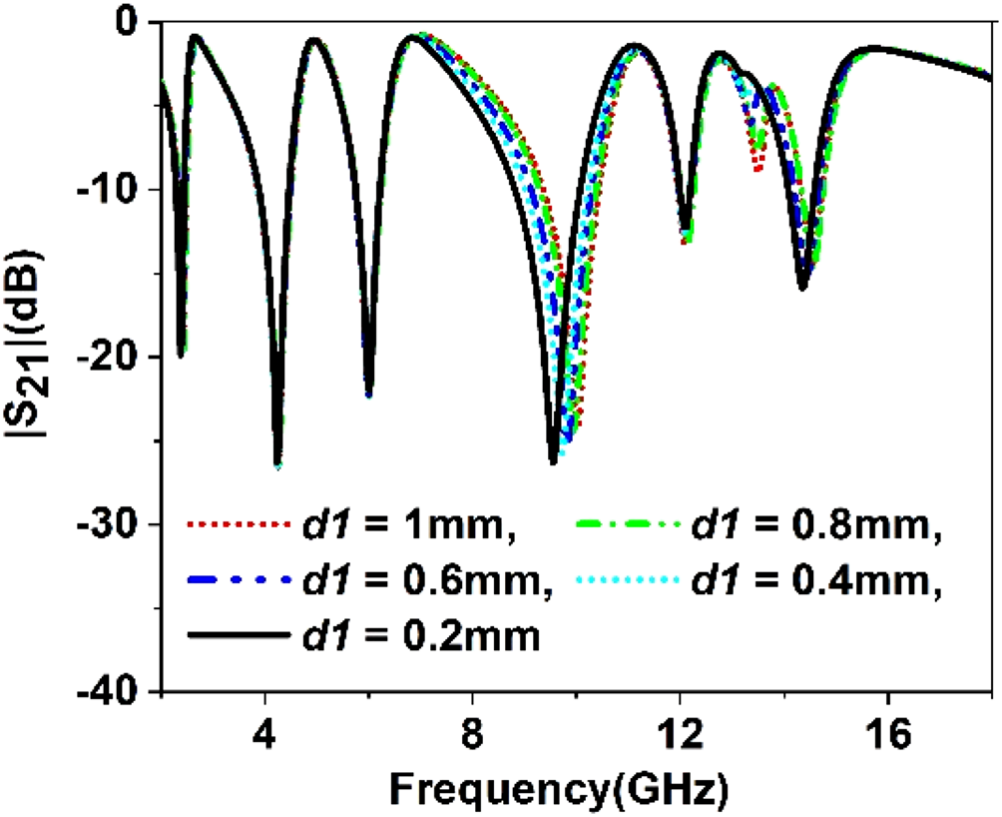
Study of variation of |S_21_| with outer ring split gap, *d1.*

**Figure 10:**
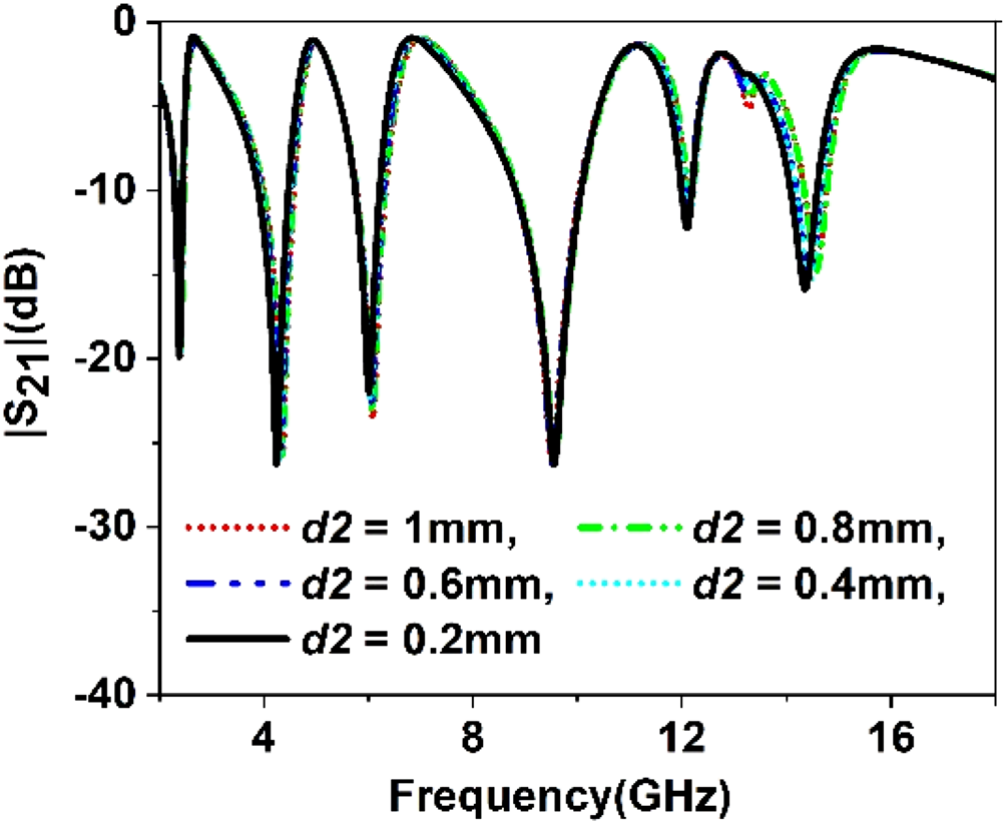
Study of variation of |S_21_| with second outer ring split gap, *d2.*

**Figure 11:**
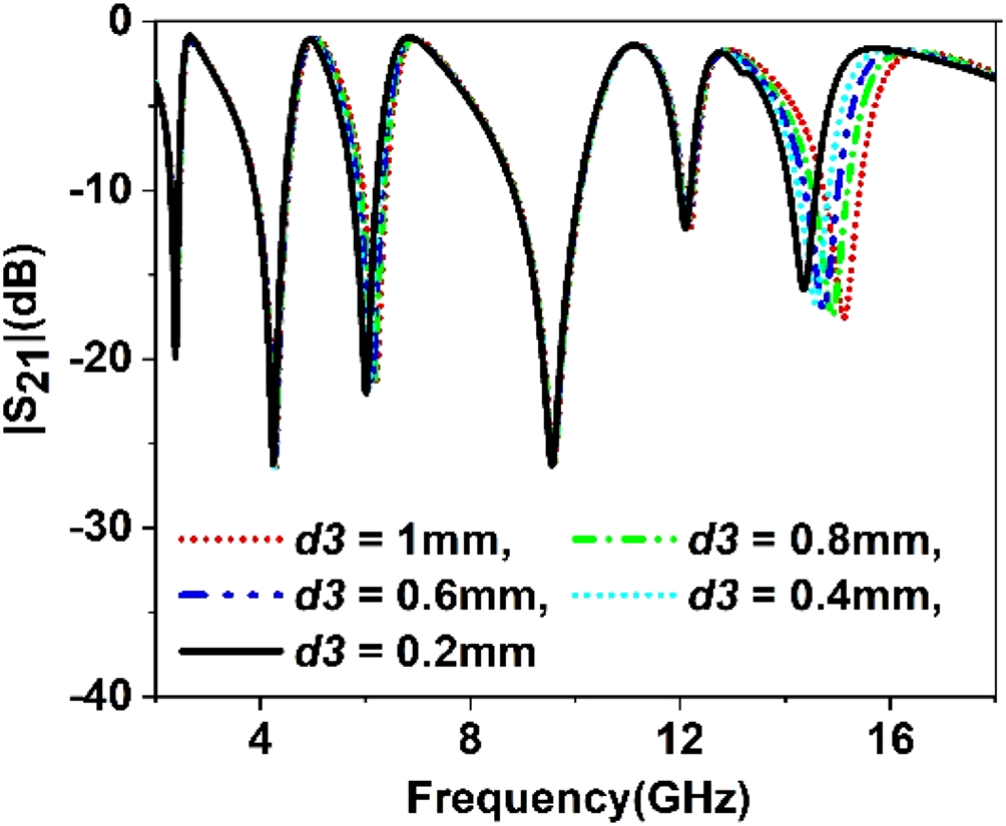
Study of variation of |S_21_| with third inner ring split gap, *d3.*

**Figure 12:**
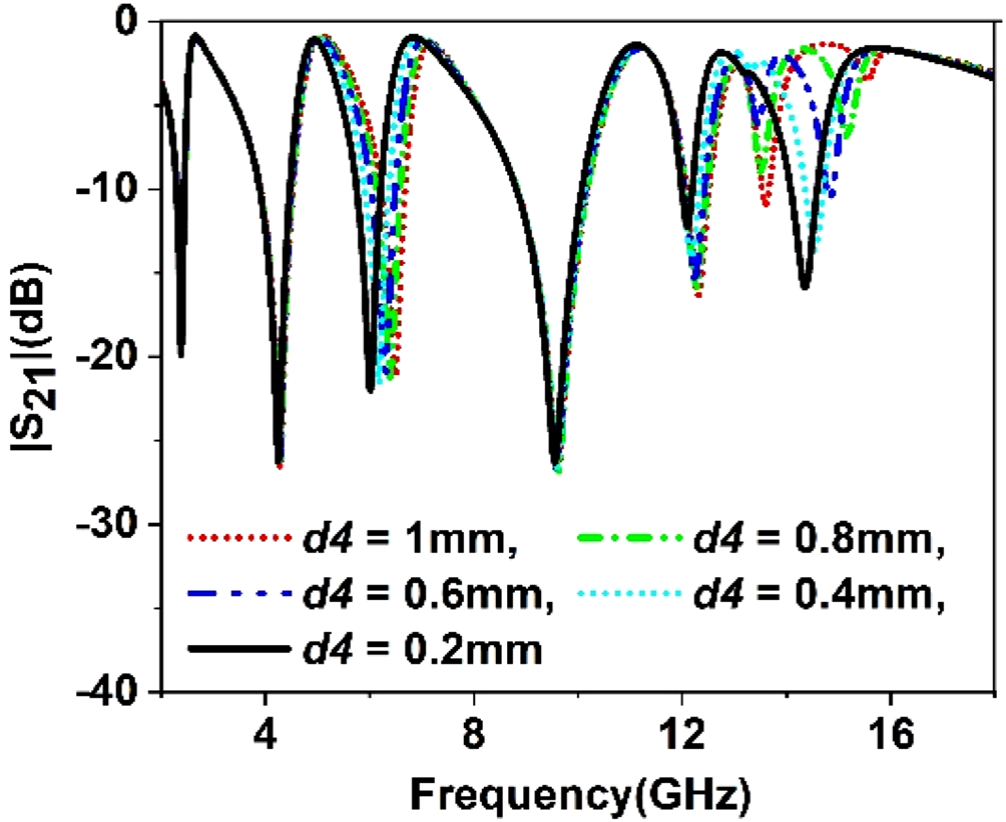
Study of variation of |S_21_| with circular ring split gap, *d4.*

**Figure 13:**
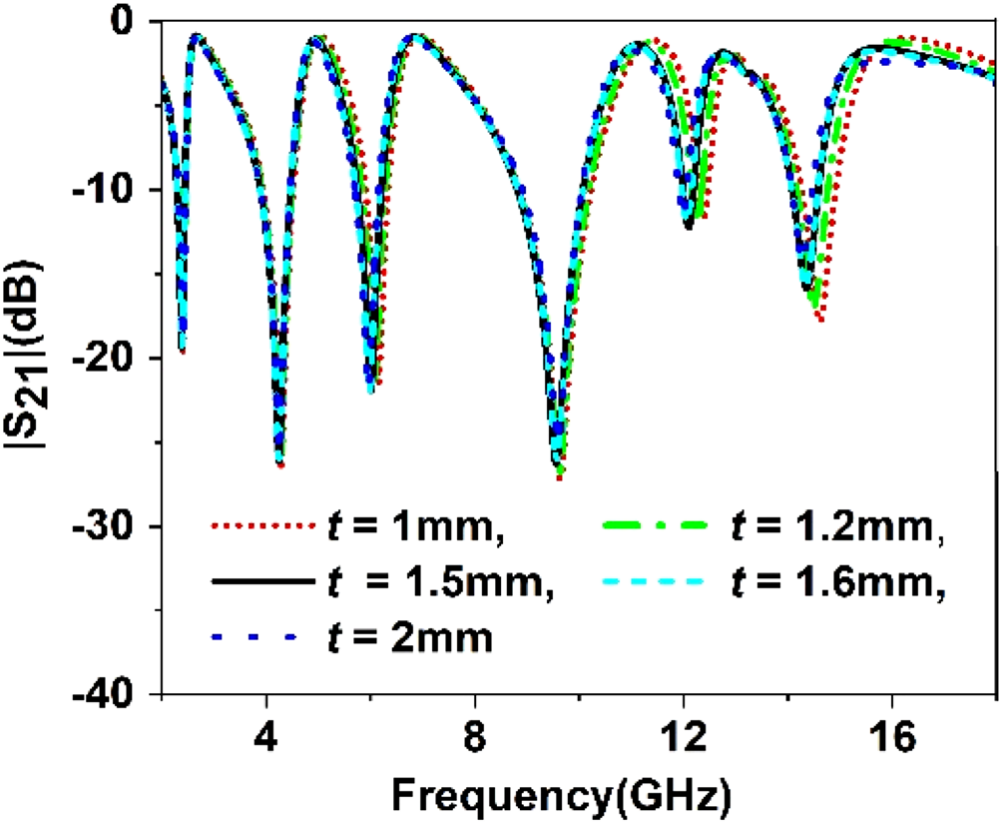
Effect of various change in substrate thickness on S_21_.

The split gap in the ring contributes to the capacitive effect, and changing the split gap modifies the capacitance formed by it. Since the ring with split gap acts as LC resonance circuit whose resonance frequency depends on the value of L and C, thus variation in the split gap has an impact on the resonances of the MTM unit cell. Similarly, substrate thickness and copper backplane also exhibit their influences on determining the characteristics of the MTM. Parametric studies have been performed in this section considering split gaps of different rings, substrate thickness, ground plane height as variables to observe the impact of parametric changes of these variables. Figure 9 represents the S_21_ for the variations of the split gap of the first ring. It is noticed that the change of split gap, *d1* of this ring, exhibits no observable effect at the first, second, third, and fifth resonances. Contrarily, it exhibits a pronounced effect at the fourth and sixth resonances. When the gap distance is changed from 1 mm to 0.2 mm, these two resonances are shifted towards the lower frequencies with an increasing magnitude. It is also visible from Fig. 9 that when *d1* is greater than 0.2 mm, another low magnitude harmonic appears around 13 GHz, whose amplitude decrease with the decreasing the split gap, *d1*. Similar effects are also noticed in Figs. 10, 11 and 12 for other split gaps d2, *d3,* and *d4* corresponding to the second, third, and fourth rings. As in Fig. 10, change of split gap, *d2* shows its impact on second, third, and last resonances where the effect is minor in second and third resonances. In Fig. 11, the influence of the third ring split gap, *d3* is observed on third and final resonances, whereas circular ring split gap, *d4* exhibits a dominant effect on third, fifth, and final resonances as presented in Fig. 12. The impact of FR4 substrate thickness is also scrutinized for several thicknesses, *t* = 1, 1.2, 1.5, 1.6, 2 mm, and outcomes are depicted in Fig. 13. A study on the effect of thickness of the substrate reveals that at a low frequency (up to 5 GHz), no significant difference in resonance is observed for various thicknesses of the substrate. Above 5 GHz, a considerable difference in resonances is observed, since resonance frequencies are shifted towards the lower value as the substrate thickness increases. However, the changes of resonance frequencies are nominal in 15, 1.6, and 2 mm.

**Figure 14:**
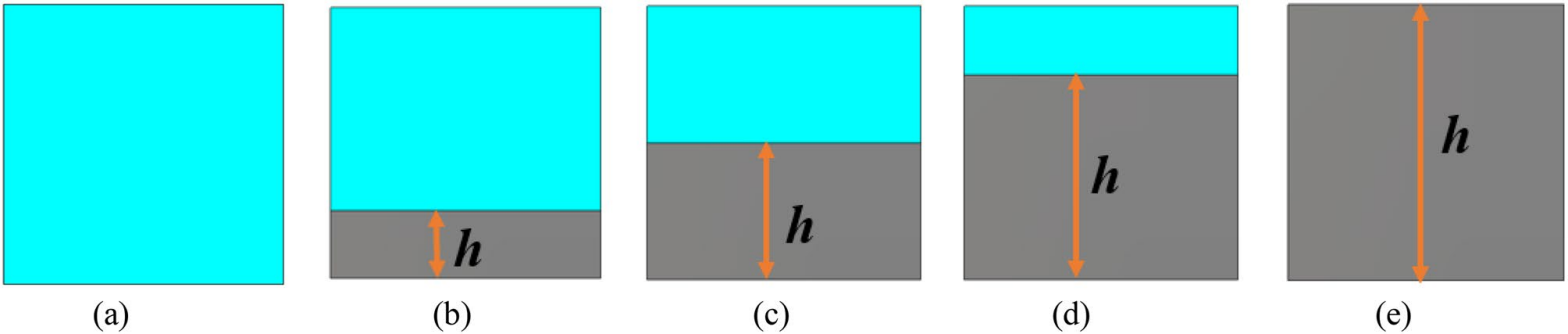
MTM unit cell with several copper back: (**a**) no copper back (*h* = 0 mm), (**b**) copper height, *h* = 2 mm, (**c**) *h* = 4 mm, (**d**) *h* = 6 mm (**e**) Full copper back (*h* = 8 mm).

**Figure 15:**
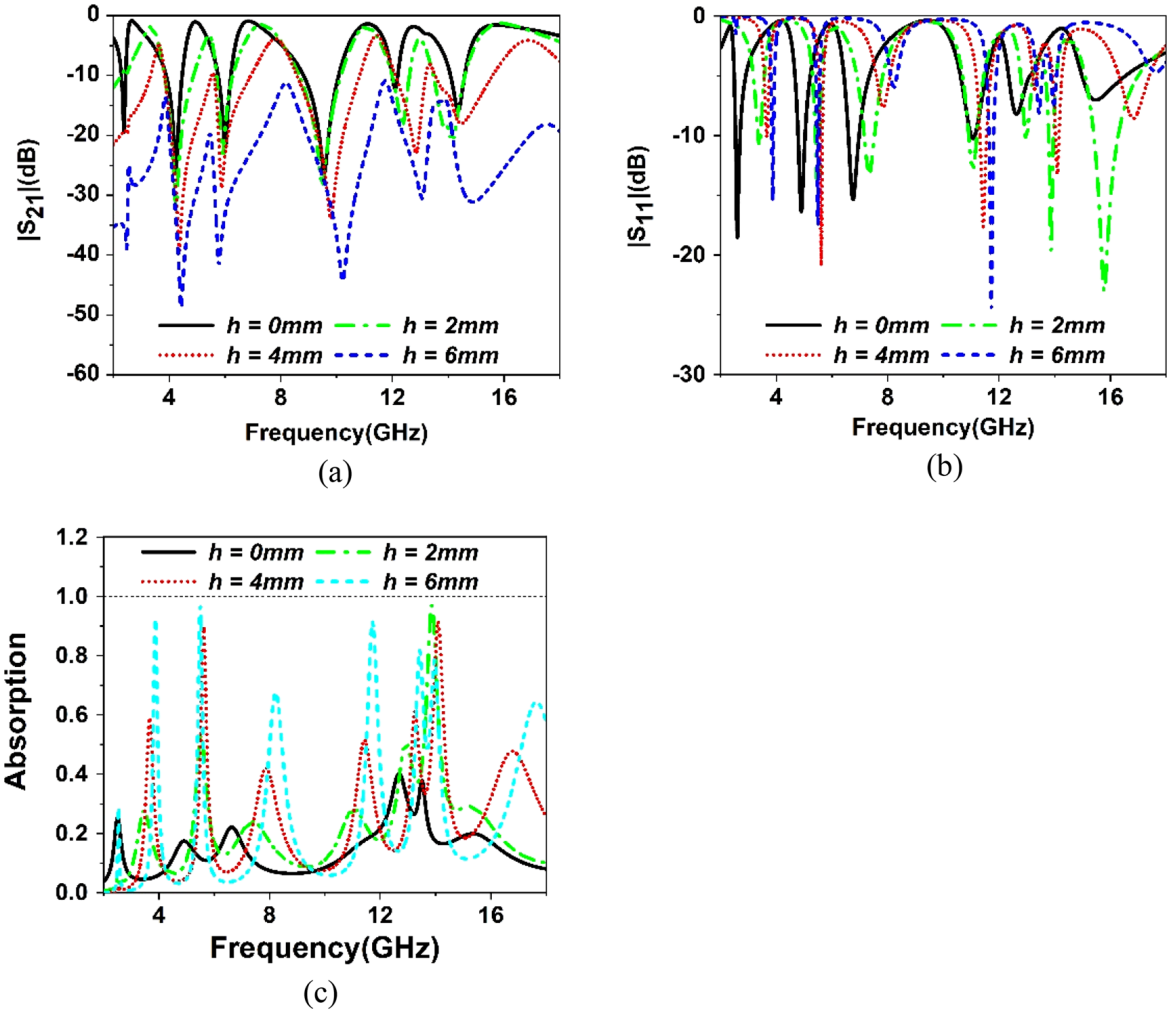
(**a**) Variation of S_21_ change of ground plane height. (**b**) Variation of S_11_ for the various ground plane of height. (**c**) Variation in absorption property with the backplane of different heights.

**Figure 16:**
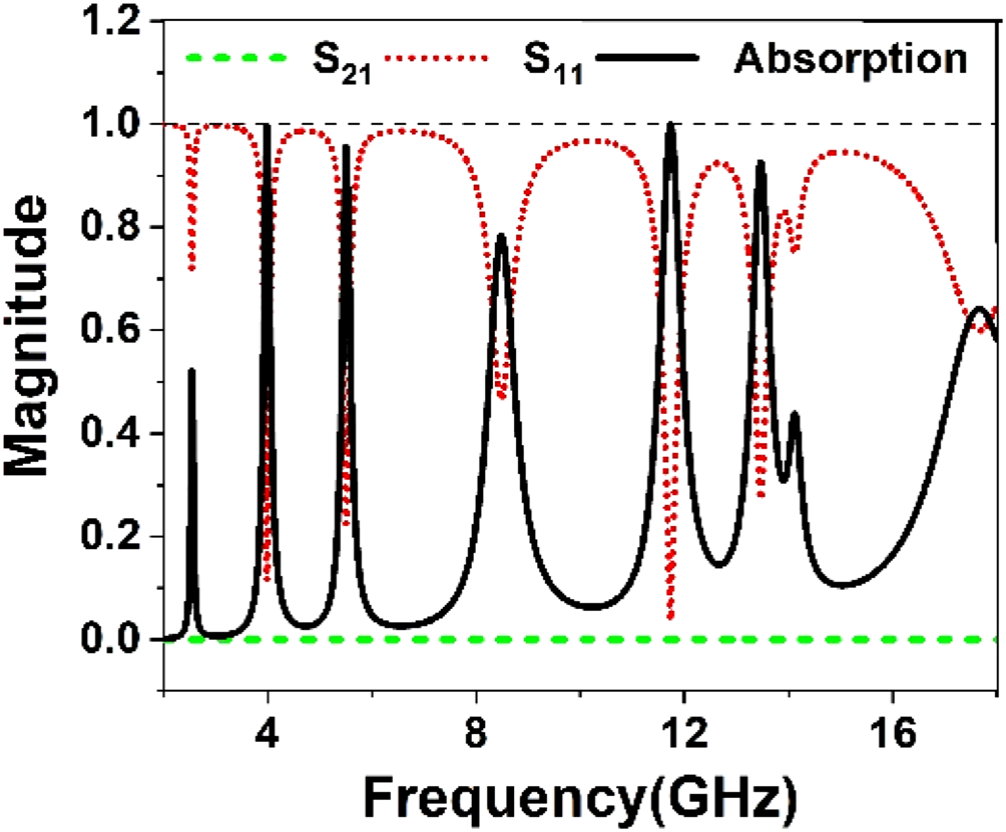
S_21_, S_11,_ and absorption plot for full copper back.

The proposed metamaterial unit cell is constructed without any copper backplane. The effect of the copper backplane on transmission and reflection coefficient is studied by using a copper backplane of different heights, as shown in Fig. 14. The transmission and reflection spectra are depicted in Fig. 15a and b. From Fig. 15a, it is observed that transmission decreases gradually as the copper height increases. It is because the copper as a conducting medium hindrance the transmission of the incident wave. The inclusion of copper in the backside introduces a current flow in the backside, which generates a magnetic field that interacts with the transmitted wave and reduces the transmission. As the copper part in the backside increases, the reduction of the transmitted wave increases gradually. When the full copper backplane is used, the electromagnetic wave transmission becomes zero, as shown in Fig. 16. An investigation of the reflection spectra shown in Fig. 15b reveals that the resonance of S_11_ shifts in frequency and magnitude with a change of copper backplane. It is also due to the reason that different copper backplane introduces different shunt capacitances formed by top resonator and copper back. Moreover, copper back also acts as an inductor and its property changes with the variation of the copper height. In association with the LC components of the top resonator, this inductor and capacitor modify resonance frequency and magnitude. With the increase of height of copper back, transmission decreases along with increase of reflection, thus the absorption property becomes significant. The amount of absorption can be calculated from the knowledge of S_21_ and S_11_ by using the Equation:

17 $${\text{Absorption}},\;A = 1 - S_{11}^{2} \left( \omega \right) - S_{21}^{2} \left( \omega \right)$$

The absorptions are obtained using this Equation for different heights of the copper backplane (h = 0, 2, 4, 6 mm) as depicted in Fig. 15c. In Fig. 15c, a gradual increase in absorption is noticed as the height of the copper back increases. It is because as the amount of conducting surface increases in the backside, the amount of transmitted wave decreases. The thickness of the copper (0.035 mm) is more than the skin depth of the transmitted electromagnetic waves. Thus, most of the transmitted wave is absorbed, and absorption increases with increasing copper height in the backplane. Eventually, when the whole of the backside is covered with the copper, transmission becomes zero and only the reflection exists, as shown in Fig. 16. Thus, absorption solely depends on the reflection coefficient, and the highest level of absorption is achieved in this situation. From Fig. 16, it is noticed that with a full copper backplane the metamaterial exhibits near-unity absorption at around 3.98 and 11.73 GHz. Moreover, above 90% absorption is observed at 5.5 and 13.47 GHz with two additional lower absorption peaks at 2.38 and 8.5 GHz. All these absorption spectra exhibit narrow bandwidth and high-quality factor, as summarized in Table 3. An absorption level lower than 20% is observed in the off-resonance frequency. All these results help to conclude that the same MTM unit cell with full copper back provides higher frequency selectivity, and it can be utilized in sensing and detecting applications^45^. The physical mechanism of the absorption can be realized with the help of surface current distribution of the top and the bottom layer at resonance frequencies. At resonances, antiparallel current flows in front and back metallic layers that eventually form the current loop and initiates magnetic dipole resonance^46^. The magnetic flux created by the opposite currents in two layers is coupled with the incident magnetic field; thus, absorption peaks are created by the magnetic resonances.

**Table 3:** Absorption property of the unit cell with full back plane.

Peak absorption frequency (*f*_*0*_) (GHz)	(%) absorption	Bandwidth (BW) (MHz)	Quality factor (Q = BW/*f*_*0*_)
3.98	99.6	140	28.4
5.5	95.7%	160	34.4
8.48	78.3%	480	17.6
11.73	99.9%	510	23
13.47	92.7	420	32

**Figure 17:**
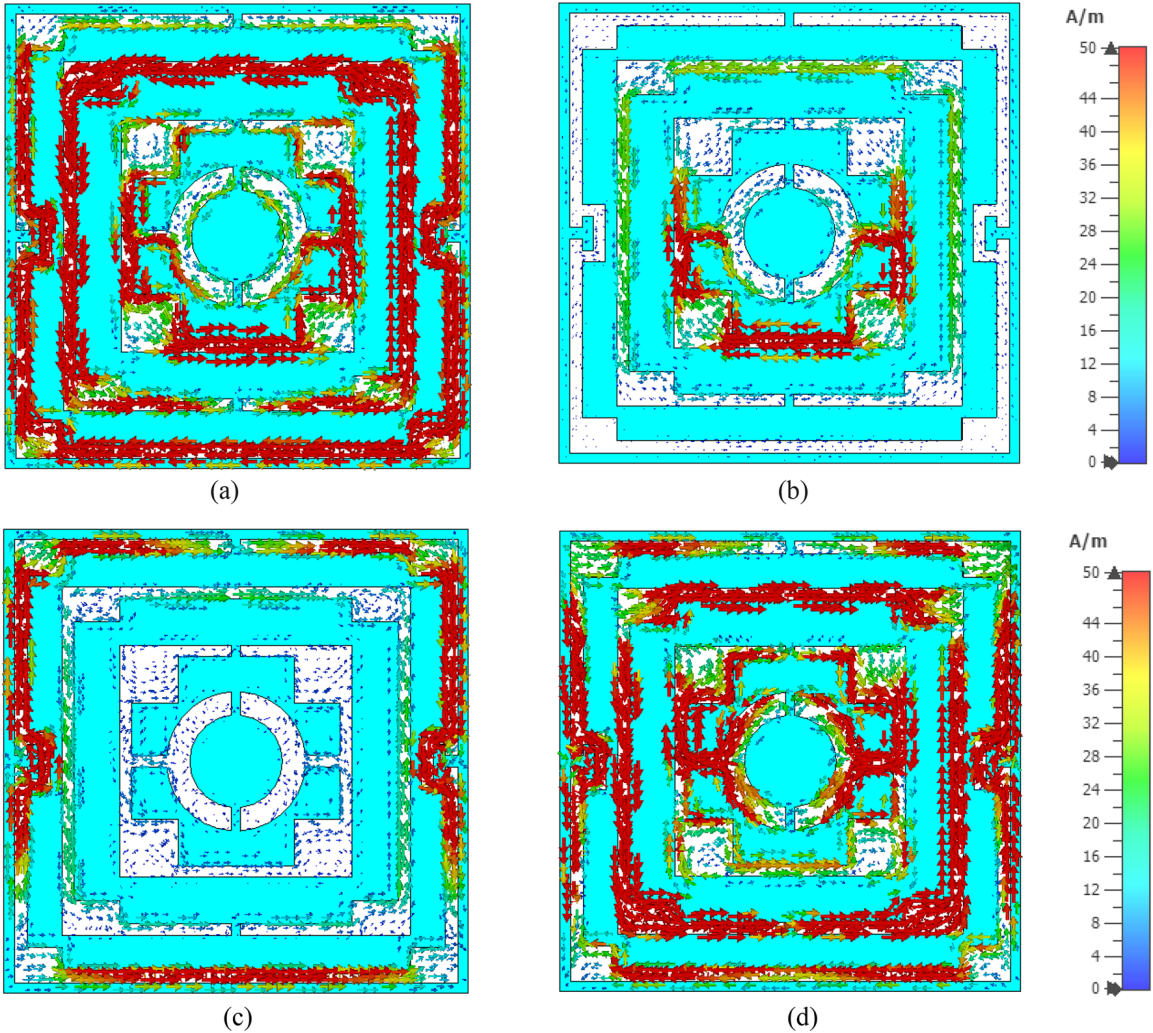
Surface current distribution at absorber resonator (**a**) 3.98 GHz (**b**) 5.5 GHz (**c**) 8.48 GHz and (**d**) 11.73 GHz.

**Figure 18:**
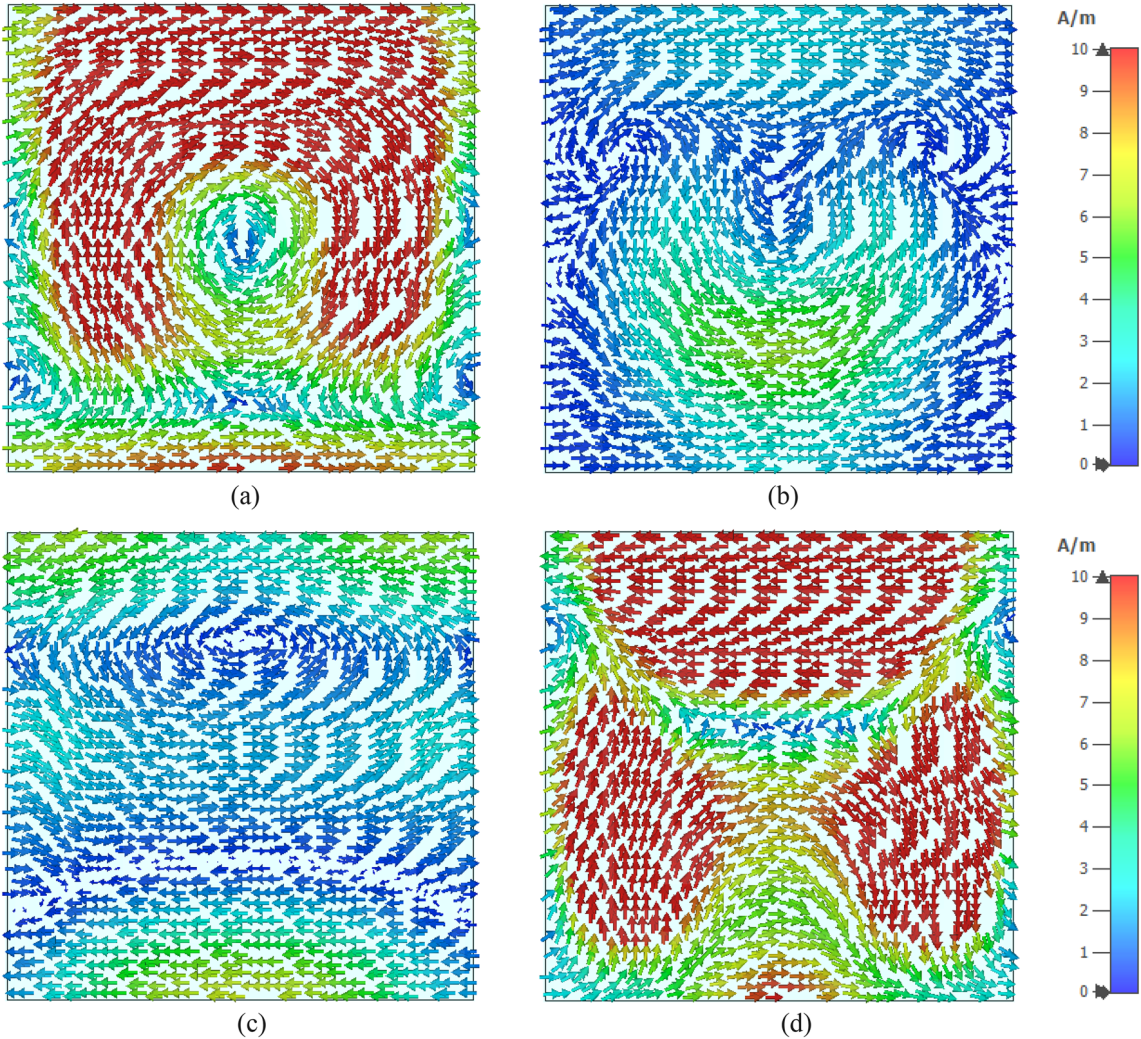
Surface current distribution at copper backplane of the resonator (**a**) 3.98 GHz (**b**) 5.5 GHz (**c**) 8.48 GHz and (**d**) 11.73 GHz.

The surface current analysis of the unit cell with the full copper backplane is conducted to understand the resonance behavior at selected resonance frequencies. The surface current patterns at the front and backside of the absorber unit cell are presented in Figs. 17 and 18, respectively, for 3.98 GHz, 5.5 GHz, 8.48 GHz, and 11.73 GHz at which the absorption peaks are observed. In Fig. 17a, a strong circular current is observed through the outer two rings and through the lower half of the interconnected two rings. The outer ring current flows in the clockwise direction, whereas the current through other mentioned rings flows anticlockwise. At the copper backplane (shown in Fig. 18a), an antiparallel current is observed below the inner rings. This antiparallel current helps create strong magnetic resonance, which is responsible for near-unity absorption at 3.98 GHz. At 5.5 GHz, a strong clockwise rotating current flows through the lower halves of the two innermost rings at the front side (shown in Fig. 17b). On the other hand, through the backplane, anticlockwise current is noticed at the same position (shown in Fig. 18b). These two antiparallel currents cause the absorption of 95.7% at this frequency. A close observation of Fig. 17c shows that current concentration is high at the outer ring, but directions of currents are opposite in vertical arms; whereas, the horizontal arms carry current in the same direction. In the backplane, noticeable antiparallel currents are observed near the two horizontal arms of the outer ring (shown in Fig. 18c). Since antiparallel current between the front and backplane is concentrated in a narrow region, absorption at this frequency is low. Contrary to this, at 11.73 GHz, significant antiparallel current is noticed (shown in Figs. 17d and 18d) at the front and back of the absorber unit cell. It is also noticed that the upper halves of the two innermost rings form a current loop with strong currents flowing in the clockwise direction. Anticlockwise rotating currents through the lower halves of the two innermost rings are also noticed in this frequency. Moreover, strong surface currents are also observed through the outer two rings. A comparison between front and backplane current indicates that antiparallel currents between these two planes are widespread. Thus maximum magnetic resonance occurs at this frequency that contributes to maximum absorption of 99.9%.

## Result and discussion

**Figure 19:**
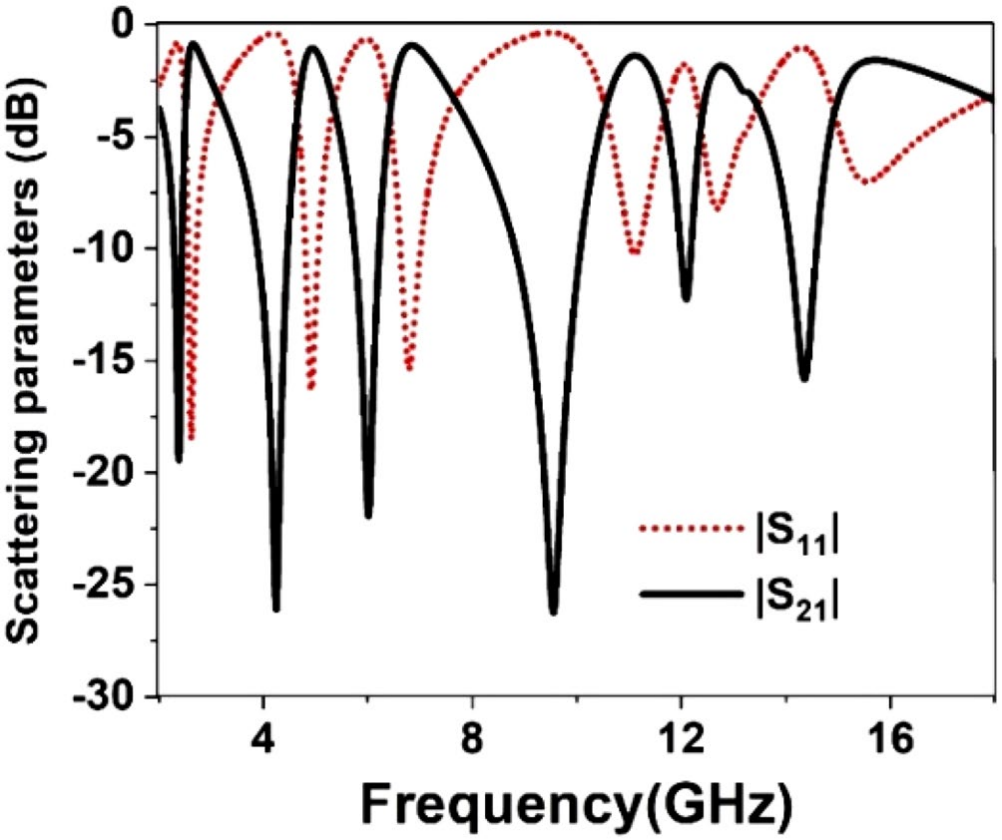
Transmission coefficient and reflection coefficient of the unit cell.

**Figure 20:**
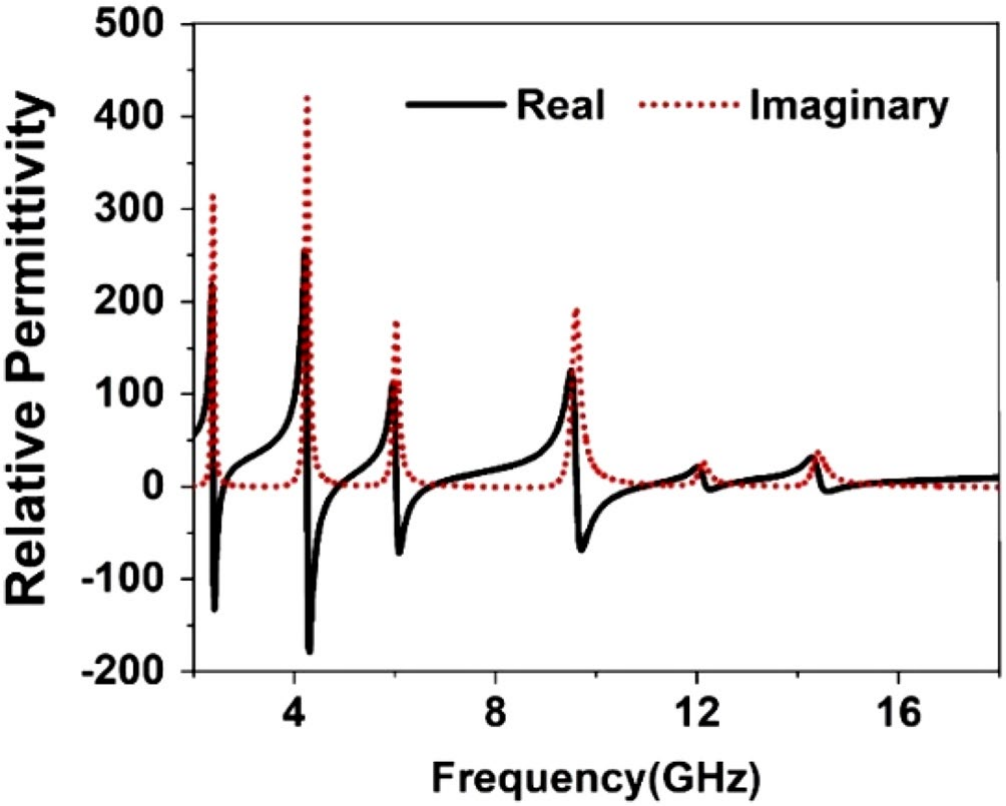
Relative permittivity, $\varepsilon_{r}$ of the proposed unit cell.

**Figure 21:**
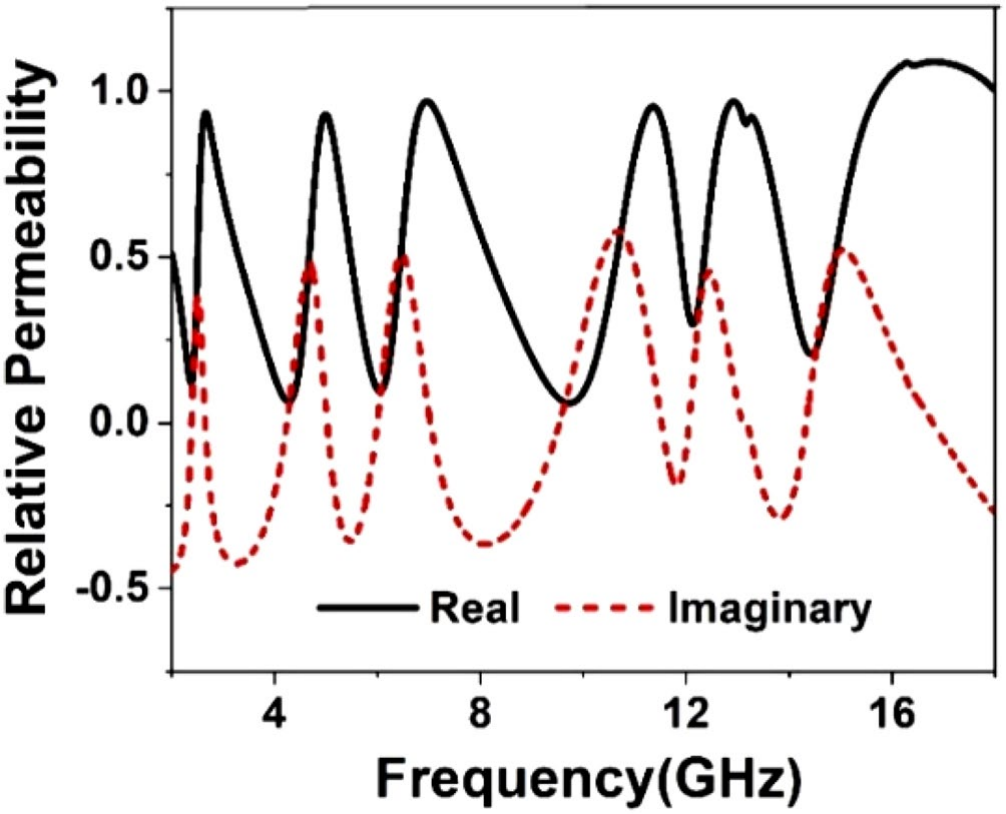
Relative permeability, $\mu_{r}$ of the unit cell.

**Figure 22:**
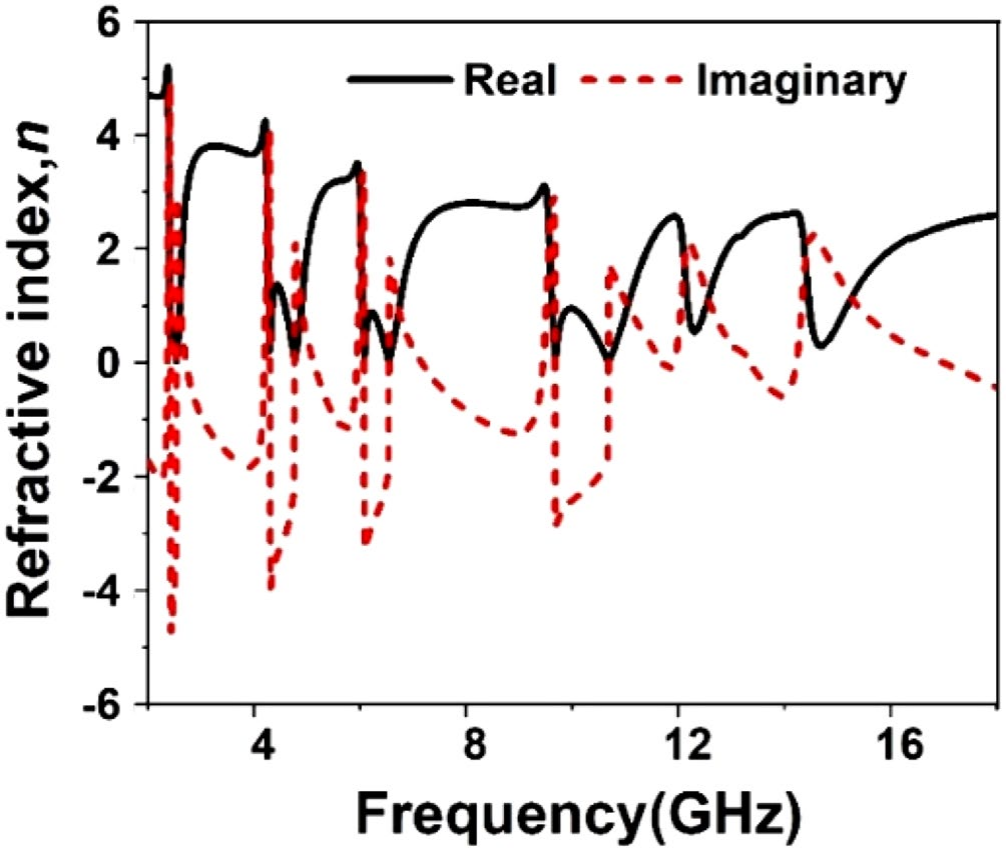
Refractive index, *n* of the unit cell.

The result of the unit cell is analyzed through simulation performed by CST, and then it is verified by comparing the simulated result with measurement one. Figure 19 shows the S_21_ and S_11_ curves expressing that there are in total of six resonances of S_21_ taking place at 2.38, 4.24, 5.98, 9.55, 12.1, and 14.34 GHz. This Figure reveals that every resonance of S_11_ occurs after the resonance of S_21;_ thus, it ensures the electrical resonances. The relative permittivity expressed in Fig. 20 exhibits negative permittivity near the resonances of the S_21_. At the same time, the effective permeability graph is represented in Fig. 21 that shows near-zero permeability at all the resonances. It is also noteworthy that relative permeability is lower than 1 with an exception above 16 GHz. Meanwhile, the refractive index plot in Fig. 22 reveals a near-zero refractive index in the negative permittivity region. The unit cell properties are summarized in Table 4, which indicates that the negative permittivity obtained within the vicinity of the resonances of S_21_. It is also noteworthy from this table that the resonances of permeability are also happened, keeping close synchronization with the negative maximum of the permittivity. Moreover, permeability is very close to zero near the resonance frequencies. The same is true in case of refractive index that shows the minimum values of 0.29, 0.02, 0.19, 0.03, 0.11, 0.036, 0.06, 0.02, 0.54 , 0.29 at 2.2, 2.52, 4.28, 4.77, 6.08, 6.54, 9.68, 10.7, 12.3 14.67 GHz respectively.

**Table 4:** Extracted parameter with important properties of the unit cell.

Parameter	Resonance/near zero Frequency(GHz)	Bandwidth(GHz)	Bandwidth threshold
S_21_	2.38, 4.24, 5.98, 9.55, 12.1, 14.34	0.14, 0.55, 0.46, 1.89, 0.2, 0.3	S_21_ < − 10 dB
$\varepsilon_{r}$	2.381, 4.28, 6.06, 9.68, 12.2, 14.53	0.21, 0.61, 0.64, 1.21, 0.26, 0.53	$\varepsilon_{r}$ < 0
$\mu_{r}$	2.38, 4.28, 6.06, 9.75, 12.15, 14.47	0.25, 0.91, 0.66, 1.75, 0.2, 0.56	$\mu_{r}$ < 0.3
*n*	2.381, 4.28, 6.08,9.68, 12.3,14.67	0.2, .07, 0.7, 1.14, 0.3, 2.7	*n* < *1*

**Figure 23:**
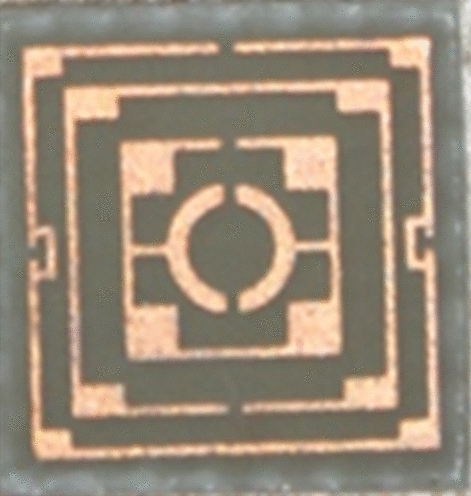
The fabricated prototype of the unit cell.

**Figure 24:**
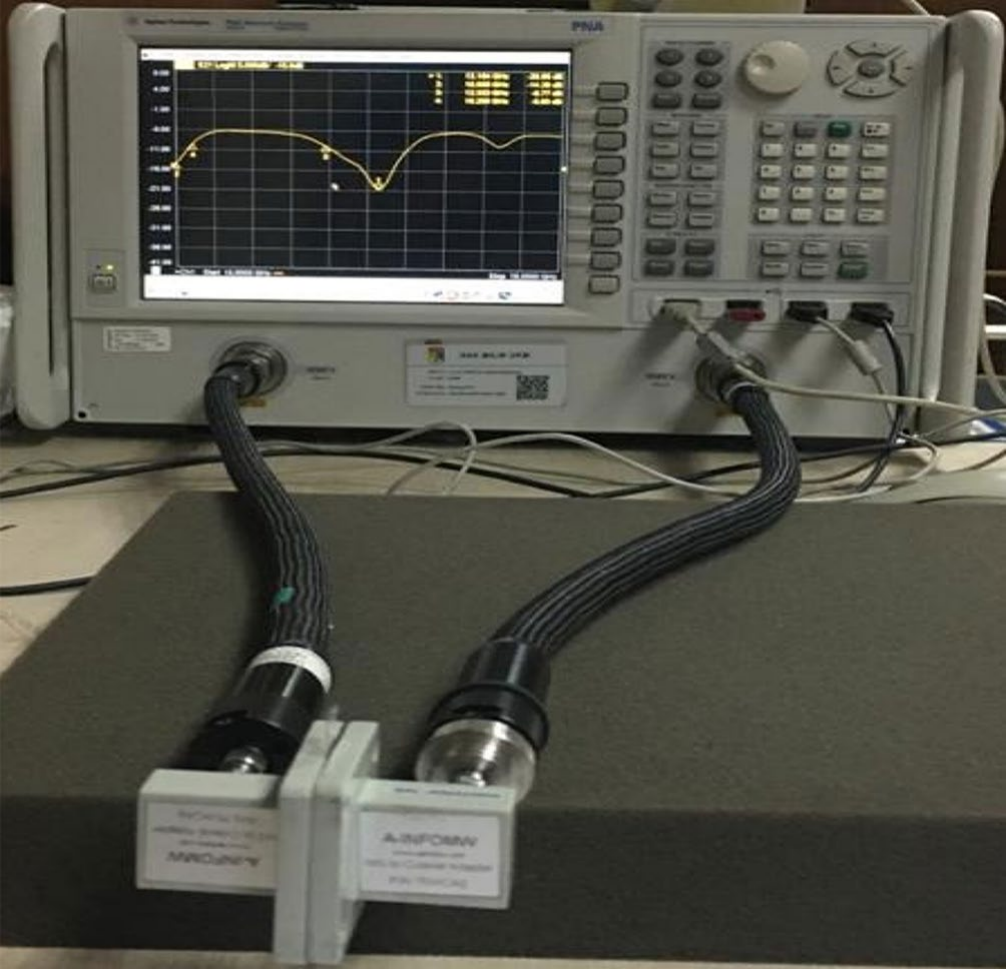
Experimental setup to measure S_21_ for the unit cell.

**Figure 25:**
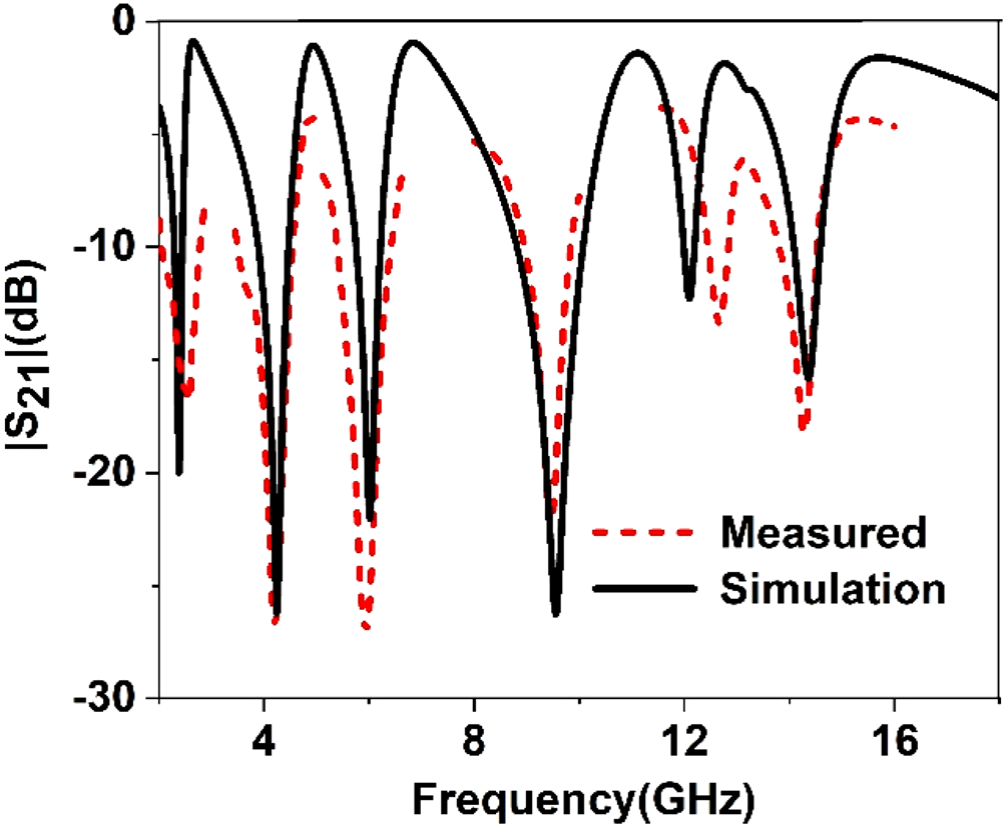
Simulated and measured S_21_ for the unit cell.

The simulated performance of the proposed metamaterial unit cell is validated by measurement. For measurement purposes, the prototype of the unit cell is fabricated that is presented in Fig. 23. The measurement setup is presented in Fig. 24, where two waveguide ports are connected to a Vector Network Analyzer(VNA) within which the unit cell is placed. The measured result is plotted in Fig. 25 with the simulated result. From this Figure, it is noticed that the measured result is well-matched with the simulated one. A small mismatch in frequency and magnitude between simulation results and measured results is observed from this Figure. The resonances around 2.38, 6 and 12.1 GHz show a frequency deviation among measured and simulated results. This shift in frequency between measured and simulated results may be due to the errors incurred during fabrication. Moreover, the mutual coupling effect between two waveguide ports along with the calibration error of VNA is also added with the measured results, which causes a difference in frequencies and magnitudes between measured and simulation results. Despite this mismatching, the measured result shows a close similarity with simulation and covers the S, C, X, and Ku band with six resonances.

**Figure 26:**
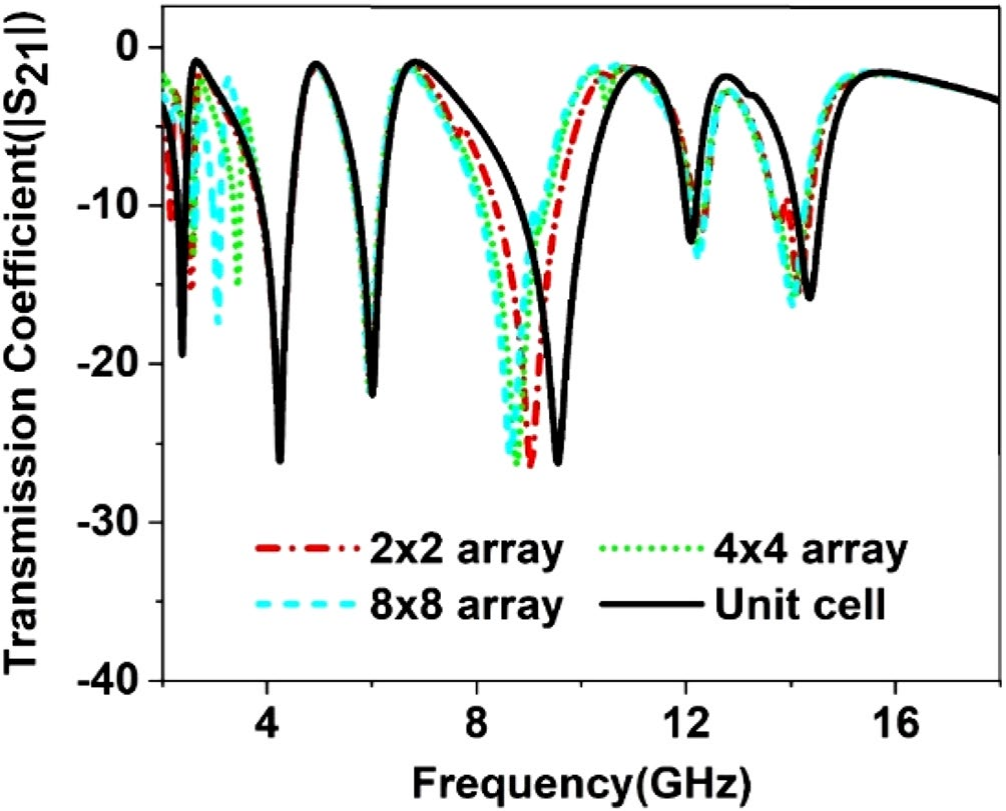
|S_21_| in dB for different arrays and unit cell.

**Figure 27:**
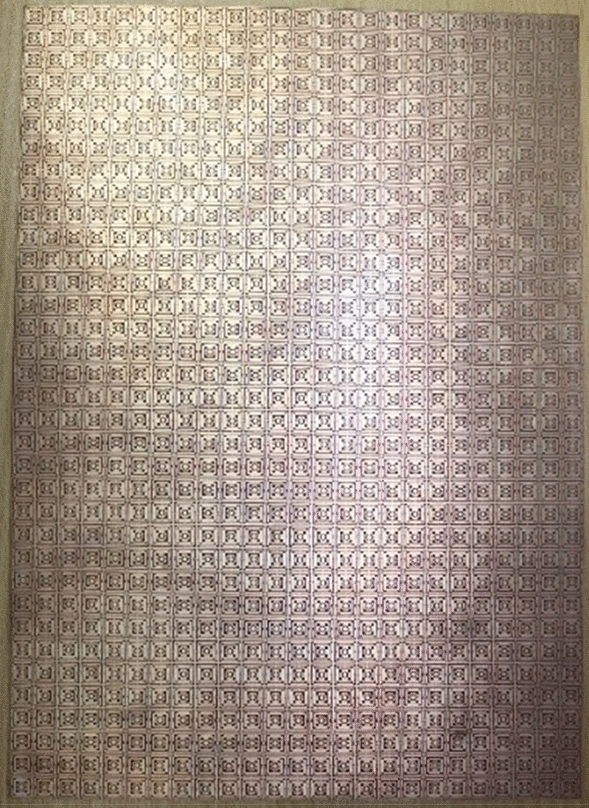
Array prototype consisting of 25 × 35-unit cells.

**Figure 28:**
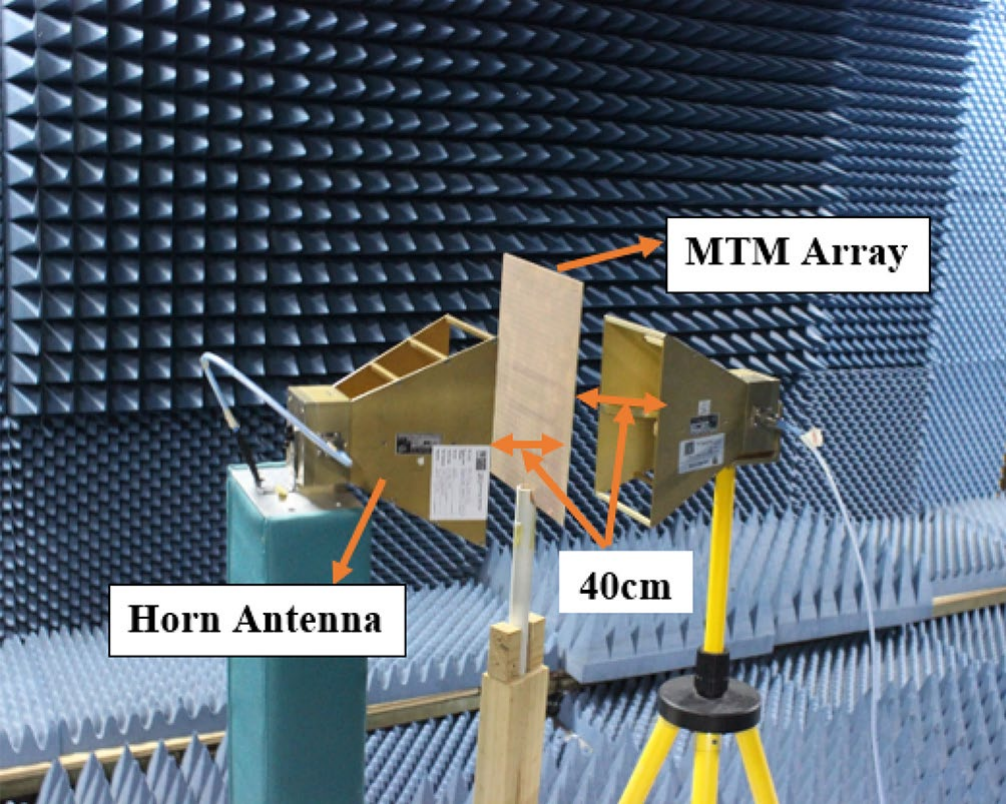
Free space array measurement setup.

**Figure 29:**
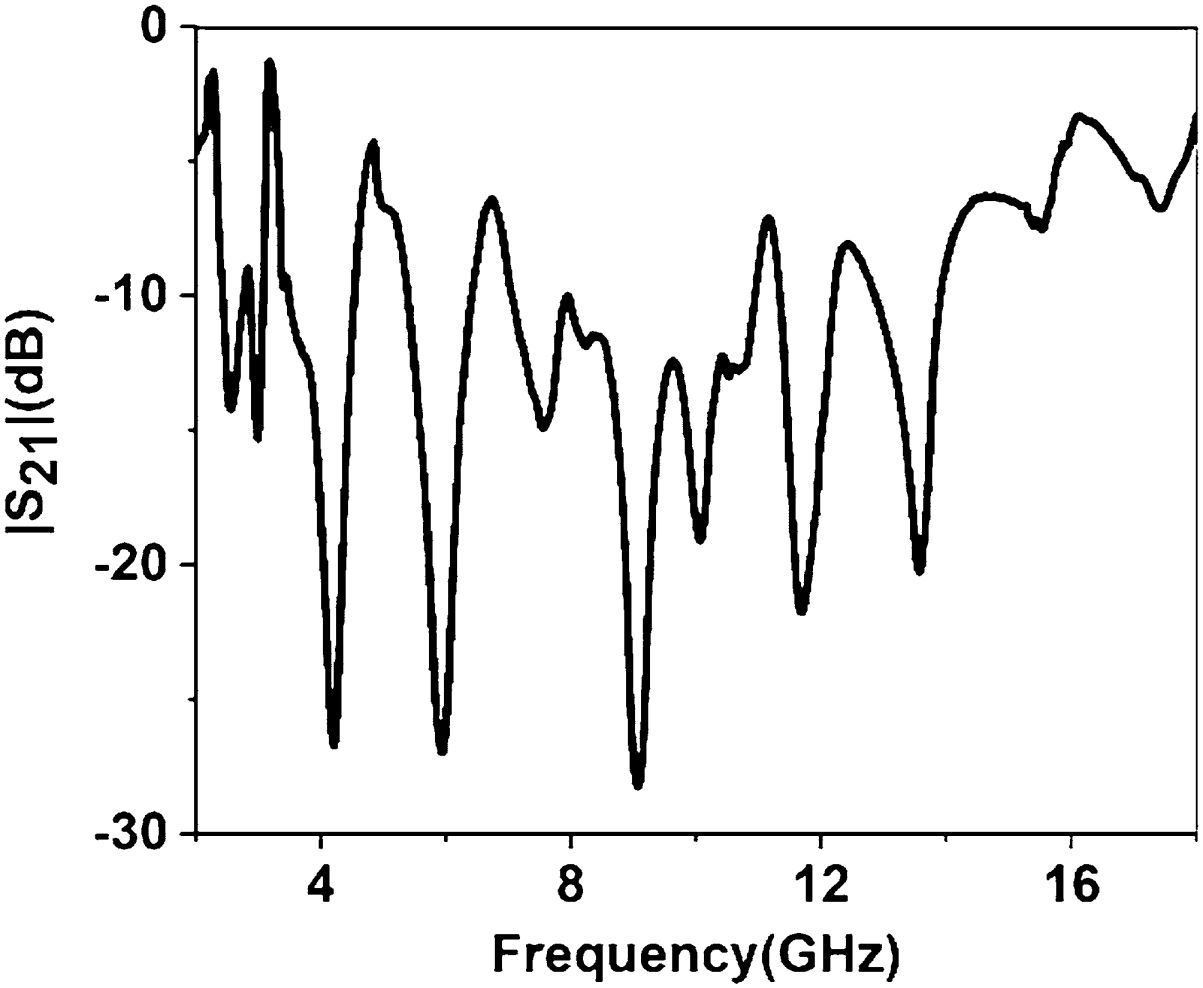
Measured |S_21_| for 35 × 25 array.

Since in many applications array of unit cells is utilized instead of a single unit cell. For this reason, array performance is also investigated considering 2 × 2, 4 × 4, and 8 × 8 arrays of the unit cells. The simulation setup is similar to the unit cell simulation presented in Fig. 2. Figure 26 represents the S_21_ response of these arrays comparing with the unit cell response. As depicted in Fig. 26, within 4–8 GHz, S_21_ responses for all arrays are nearly unaffected compared with the unit cell. Unlikely, within 2–4 GHz and 8–16 GHz, the array responses differ from unit cell response. For the 2 × 2 array, two resonances occur within 2–4 GHz at 2.15 GHz and 2.56 GHz instead of single resonance at 2.38 GHz for the unit cell. Within 8–16 GHz, no harmonics are observed, but the resonance frequency deviation is more pronounced. The resonances around 9 and 14 GHz shift towards lower frequency, whether the resonance around 12 GHz shifts towards right. Meanwhile, 4 × 4 exhibits two resonances at 2.5 and 3.4 GHz, which deviate more towards high frequencies. Similar shifting in resonance frequencies also occurred in the resonances within 8–10 GHz. Noteworthy to mention that, frequency shifting behavior is also noticeable in the array constituting 8 × 8 cells, as shown in Fig. 26. The resonance frequencies of each array with percentage deviations compared to corresponding unit cell resonances are listed in Table 5. In Arrays, the S and X band's resonances undergo more shifts in frequency, whereas the Ku band is moderately susceptible to change in frequency, and the C band is less prone to change. But this deviation is not more than 10%, and every array covers four bands with six resonances. It is noticeable that each array generates a harmonic in S-band, which is at 2.16, 3.44, and 3.1 GHz for 2 × 2, 4 × 4, and 8 × 8 arrays, respectively. The array performance is also studied by experiment with the use of a 25 × 35 array (shown in Fig. 27) that provides a larger footprint compared to the wavelength of the desired frequency bands. The free space measurement method is implied for array performance investigation, where the experimental setup is arranged in an anechoic chamber, as shown in Fig. 28. Two horn antennas are used, one for transmitting and another for receiving the transmitting signal. The array dimension of 200 × 280 mm^2^ is larger compared to the aperture size of the horn antenna, and the array is placed 40 cm away from each antenna to ensure the far-field radiation distance of the antenna. The measured result is presented in Fig. 29. Multiple resonances are observed in array measurements covering the expected S, C, X, and Ku-bands. From this Figure, it is also observed that harmonics are presented in S-band. Similar harmonics are also presented in other bands with a small amount of noise. The resonance frequencies are a little bit shifted with the magnitude variation. The mutual coupling effect among the unit cells of the array is the one reason for the harmonics and frequency and magnitude shifting. Moreover, some amount of noise is also added due to the long-extended cable from the horn antenna to VNA that causes to mismatching the results compared to the other simulated results. Despite all these effects, the array provides better performance that can be utilized for the desired S, C, X, and Ku-band applications.

**Table 5:** S_21_ comparison between array and unit cell.

Band	Resonance Frequency (GHz)				% deviation of the frequency		
	Unit cell	2 × 2 array	4 × 4 array	8 × 8 array	2 × 2 array	4 × 4 array	8 × 8 array
S	2.38	2.16, 2.56	2.6, 3.44	2.6, 3.1	− 10, 6.7	8.3,	8.3
C	4.24, 6	4.24, 5.98	4.24, 5.95	4.24, 5.97	0, − 0.4	0, − 0.8	0, − 0.5
X	9.6	9.03	8.8	8.64	− 5.9	− 8.3	− 10
Ku	12.1, 14.4	12.29, 14.16	12.24, 14.06	12.2, 14	1.6, − 1.7	1.2, − 2.38	0.8, 2.8

A minus sign in % deviation indicates shifts of resonance towards lower frequency, whereas positive deviation indicates a shift towards higher.

The compactness of the dimension is also an important factor for many applications. The compactness is determined by using the effective medium ratio (EMR) that is calculated with the help of wavelength at the lowest resonance frequency, $\lambda_{0}$ and a maximum dimension of the unit cell, L: EMR = $\frac{{\lambda_{0} }}{L}$. The maximum dimension of the proposed unit cell is 8 mm, and the wavelength at 2.38 GHz is 126 mm. Thus, the EMR of this unit cell is 15.75, which ensures a compact dimension of the unit cell. It also fulfills metamaterial criteria,$\lambda_{0} /10$ (~ 12.6 mm) as the dimension is 8 cm. This compact size MTM in an array is utilized as a superstrate to enhance the gain of the antenna. The application of this MTM is more elaborately discussed in the next section. A comparison of the proposed MTM unit cell is made with other recently published states of the art and tabulated in Table 6. The comparison is made based on dimensions, number of resonances, covering bands, EMR, and applications.

**Table 6:** Performance comparison of proposed MTM with other states of the art based on number of resonances, covering band, EMR, and applications.

Ref	Year	Electrical dimension (λ × λ)	No. of resonances	Covering bands	EMR	Applications
^47^	2020	0.076λ × 0.076λ	4	C, S and X-bands	13.6	Microwave (proposed)
^21^	2020	0.116λ × 0.116λ	2	X and S-bands	8.4	Microwave (proposed)
^22^	2019	0.125λ × 0.125λ	1	C-band	8	Microwave (proposed)
^30^	2020	0.124λ × 0.1244λ	3	C, X and Ku-bands	8.03	Satellite communications (proposed)
^27^	2019	0.256λ × 0.256λ	1	S-band	3.95	Glucose sensing
^24^	2020	0.373λ × 0.825λ	1	X-band	1.2	Liquid chemical detection
^20^	2020	0.05λ × 0.085λ	1	C-band	11.75	Improve antenna gain
^48^	2020	0.35λ × 0.35λ	2	K-band	2.86	Bandwidth improvement of MIMO antenna
Proposed	2020	0.063λ × 0.063λ	6	S, C, X, and Ku-bands	15.75	Gain enhancement of the multiband antenna

## Application: gain enhancement of tapered log periodic dipole(TLPD) antenna using an array of proposed MTM

**Figure 30:**
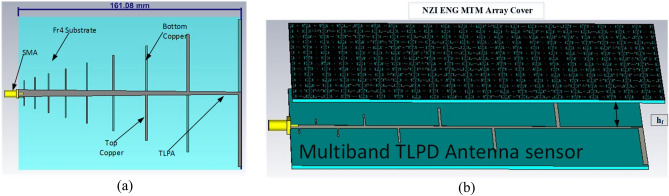
Designed TLPD Antenna with top and bottom copper (**a**) without NZI ENG metamaterial (**b**) with NZI metamaterial cover.

**Figure 31:**
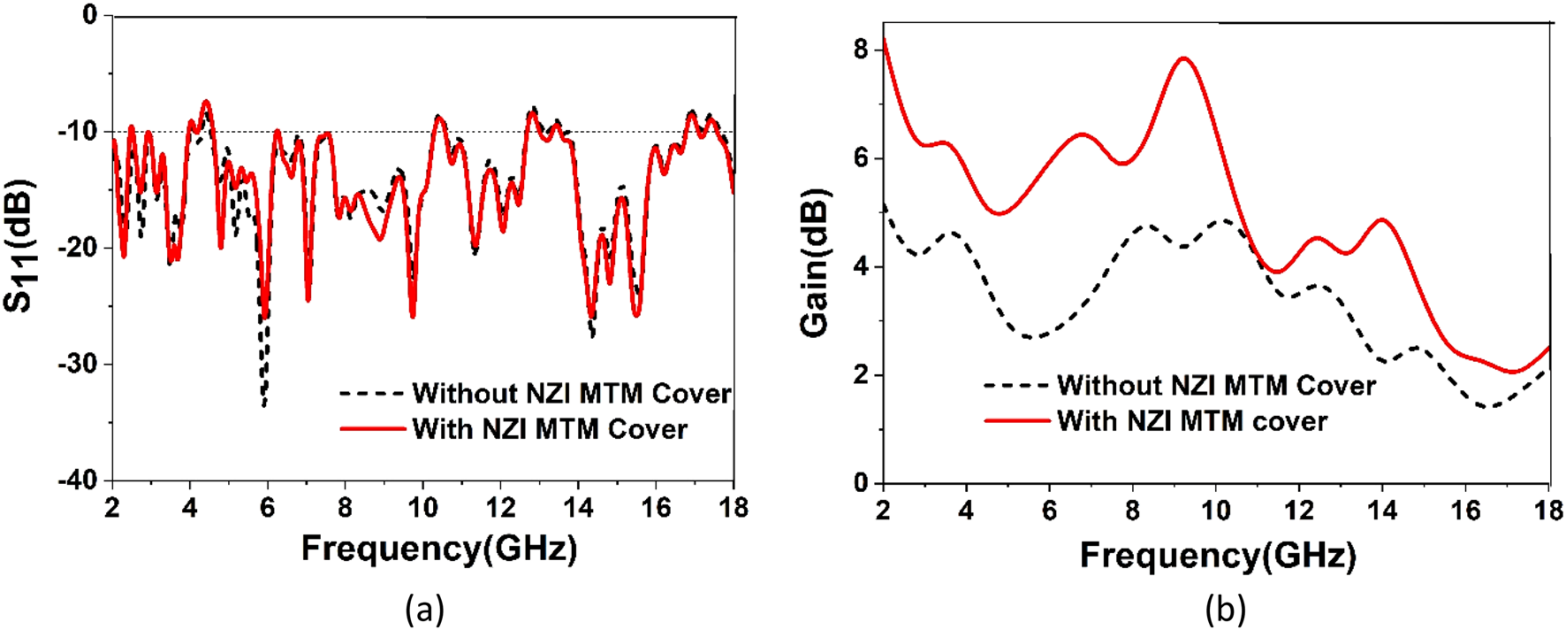
Antenna performance without and with MTM cover (**a**) Reflection coefficient (**b**) Gain.

**Figure 32:**
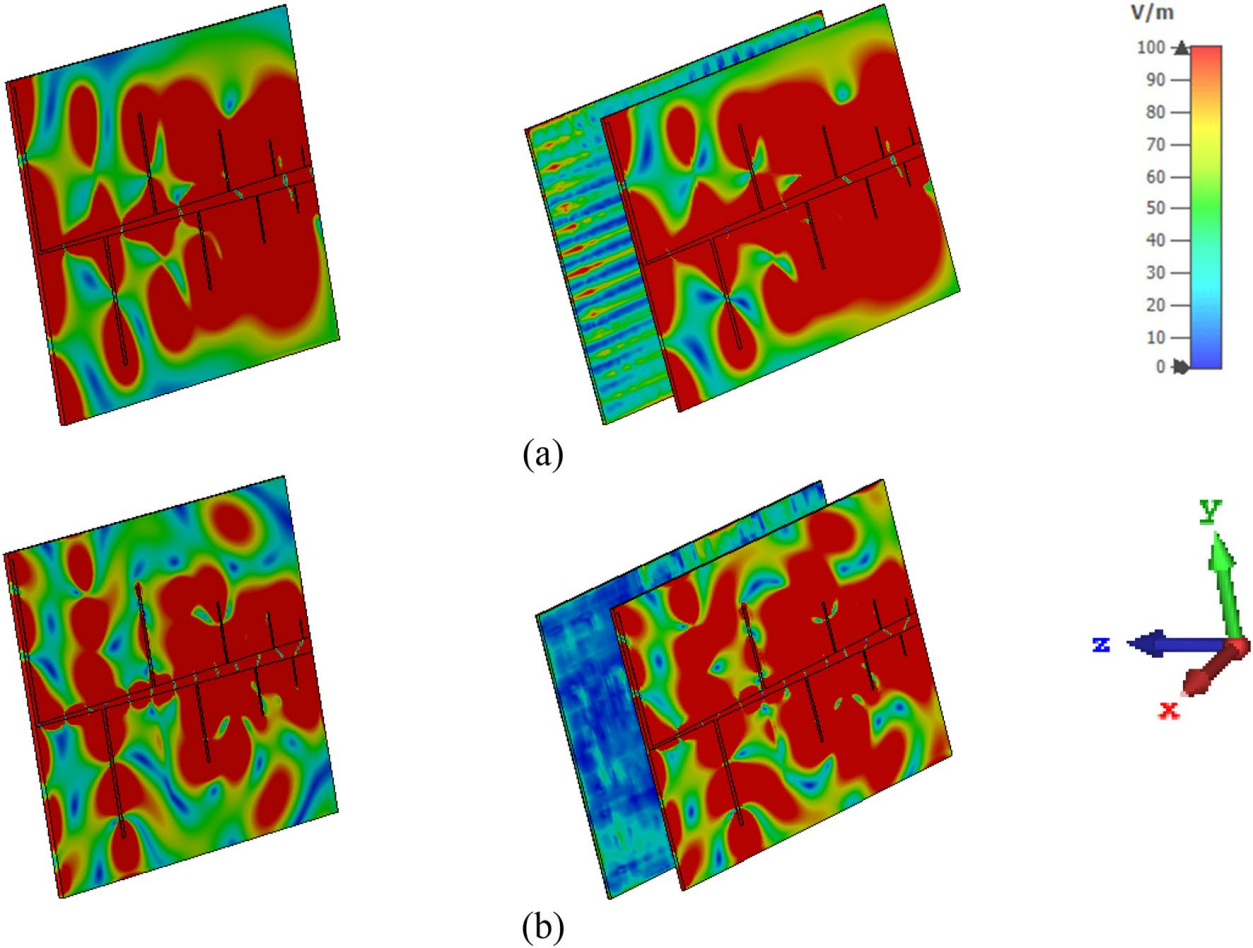
The absolute value of E-field distribution (|E|) of TLPD antenna without and with MTM cover (**a**) 2.38 GHz (**b**) 6.08 GHz.

**Figure 33:**
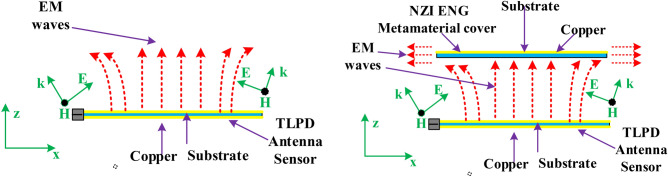
TLPD Antenna gain enhancement mechanism (**a**) Without NZI MTM Cover (**b**) With NZI MTM cover.

**Figure 34:**
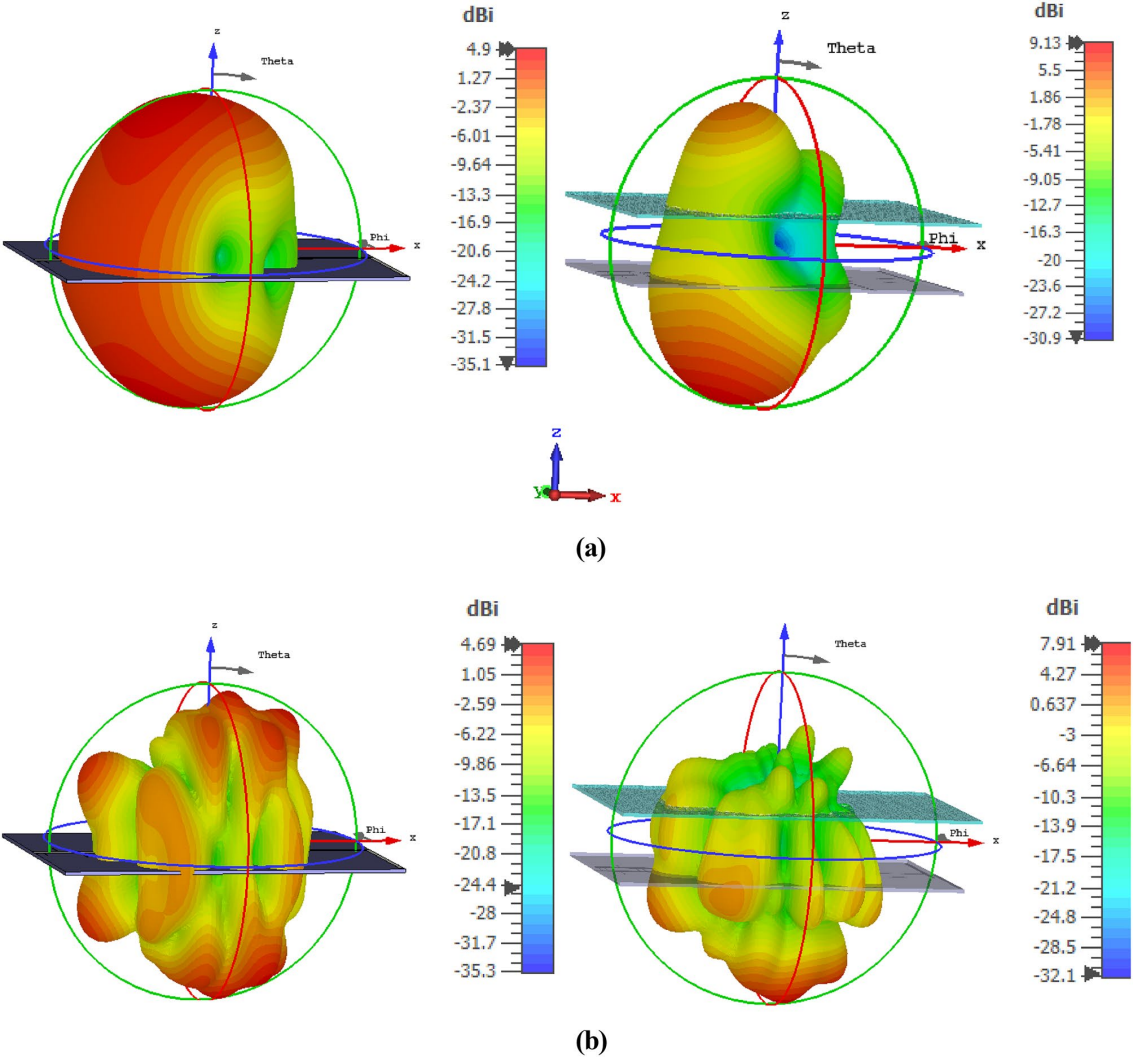
The far-field 3D radiation pattern of with and without NZI MTM cover of the TLPD antenna (**a**) 2.38 GHz and (**b**) 6.08 GHz.

As discussed above, the proposed MTM exhibits negative permittivity along with near-zero permeability and refractive index. The properties have immense applications in the field of the antenna for improving performances, especially the NZI property can be used to boost antenna gain^49,50^ and directivity^51^. For the proposed multiband ENG metamaterial application, a modified log periodic patch antenna has been designed named tapered log periodic dipole(TLPD) antenna^52^ for microwave sensing purposes in imagining. The schematic layout of the antenna is depicted in Fig. 30a. The TLPD antenna is designed on FR4 substrate with dielectric constant 4.4 and tangent loss 0.02. It consists of 9 arms at each side of the substrate, where all arms are connected with tapered copper. The obtained reflection coefficient and gain of this antenna are depicted in Fig. 31. The proposed antenna exhibits a wideband reflection coefficient though the obtained gain is very low. This low gain is not suitable for the application as a microwave-based imaging sensor. For that reason, the designed multiband MTM is used as a cover with TLPD antenna to increase the gain. The geometric layout of the antenna with MTM cover is displayed in Fig. 30b, where an array of 21 × 14 unit cells of the proposed MTM is used at a distance of 30 mm. This distance is optimized by numerous simulations so that better gain can be achieved. Figure 31a indicates that the proposed antenna with MTM provides the same bandwidth compared to without MTM covering all frequency bands under consideration. Owing to the MTM canopy over the TLPD antenna, the gain increases tremendously, as shown in Fig. 31b. It provides the maximum gain enhancement by 3.5 dB with a maximum gain above 8 dB. A higher gain over the frequency range 2–18 GHz with an average gain of 5 dB (~ 3 dB without MTM array) is also observed at about 73% more. A part of the radiated signal goes to the z-direction reducing the horizontal gain when the antenna without metamaterial is radiated, and a lower gain is noticed in that case, as in Fig. 31b. Field distribution in the horizontal plane is increased when the NZI metamaterial canopy is applied at 30 mm above the antenna. Figure 32 exhibits the antenna's electric field distribution with and without metamaterial at 2.38 and 6.08 GHz. By investigating these E field distribution, it can be expressed that in the case of MTM cover, the field is more intense and widespread in the horizontal plane of the antenna compared to without MTM. The improvement of gain and change of field distribution can be explained with the help of Fig. 33, in which sideward radiated waves are shown without and with MTM cover. As in Fig. 33a, the E field component perpendicular to the MTM cover propagates directly; but with MTM cover due to NZI characteristics, when the EM wave passed through the MTM cover, a portion of the waves becomes horizontal i,e. parallel to the XOY plane as shown in Fig. 33b. Thus, MTM cover helps to increase horizontal electromagnetic wave, which in turn enhance horizontal gain due to the change of direction of the main beam^53^. 3D far-field radiation pattern is presented in Fig. 34 at 2.38 GHz and 6.08 GHz with and without NZI MTM cover of the TLPD antenna. The radiation patterns are more directional with increased gain for the antenna with NZI MTM cover compared to the bare TLPD antenna. It is also noticed that the radiation pattern is more orientated in the horizontal plane with significant gain improvement in case of antenna with NZI MTM cover. Without NZI MTM cover, at 2.38 GHz and 5.99 GHz, the gain is 4.13 dB and 4.55 dB, respectively whereas in case of NZI MTM cover maximum directional gain is 9.13 dB and 7.89 dB, respectively.

**Figure 35:**
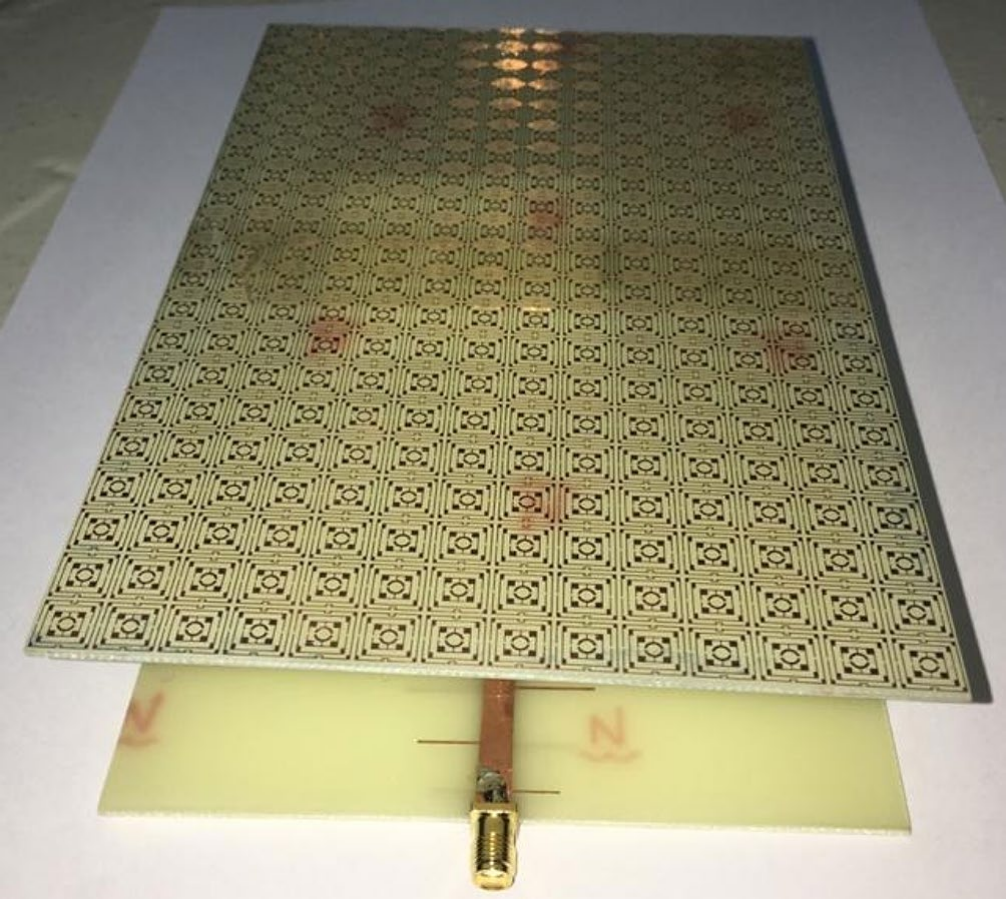
Fabricated antenna and metamateria

**Figure 36:**
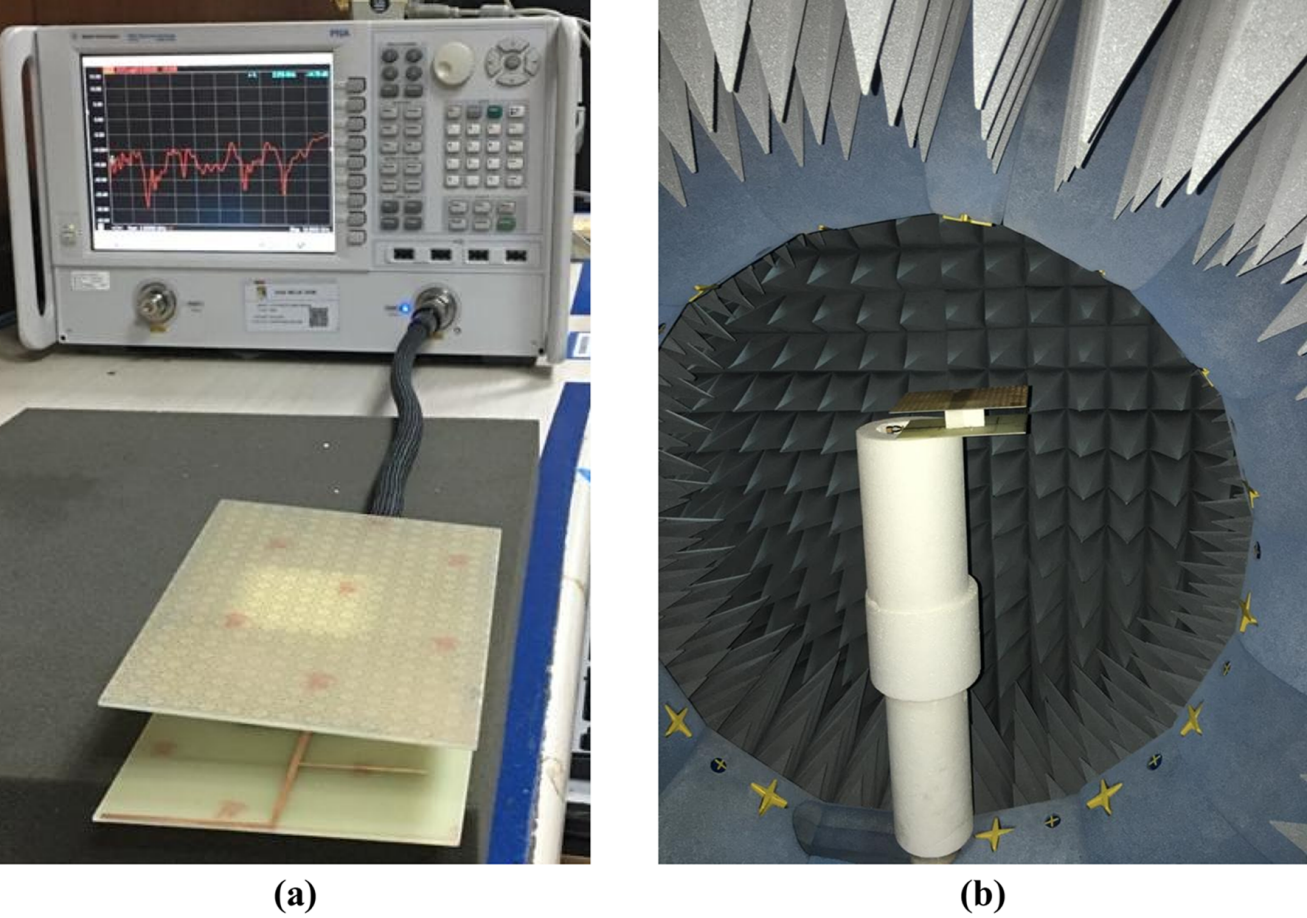
(**a**) Measurement set up for S_11_ and VSWR using VNA (**b**) Measurement setup for gain, efficiency, and radiation pattern using SATIMO near field measurement system.

**Figure 37:**
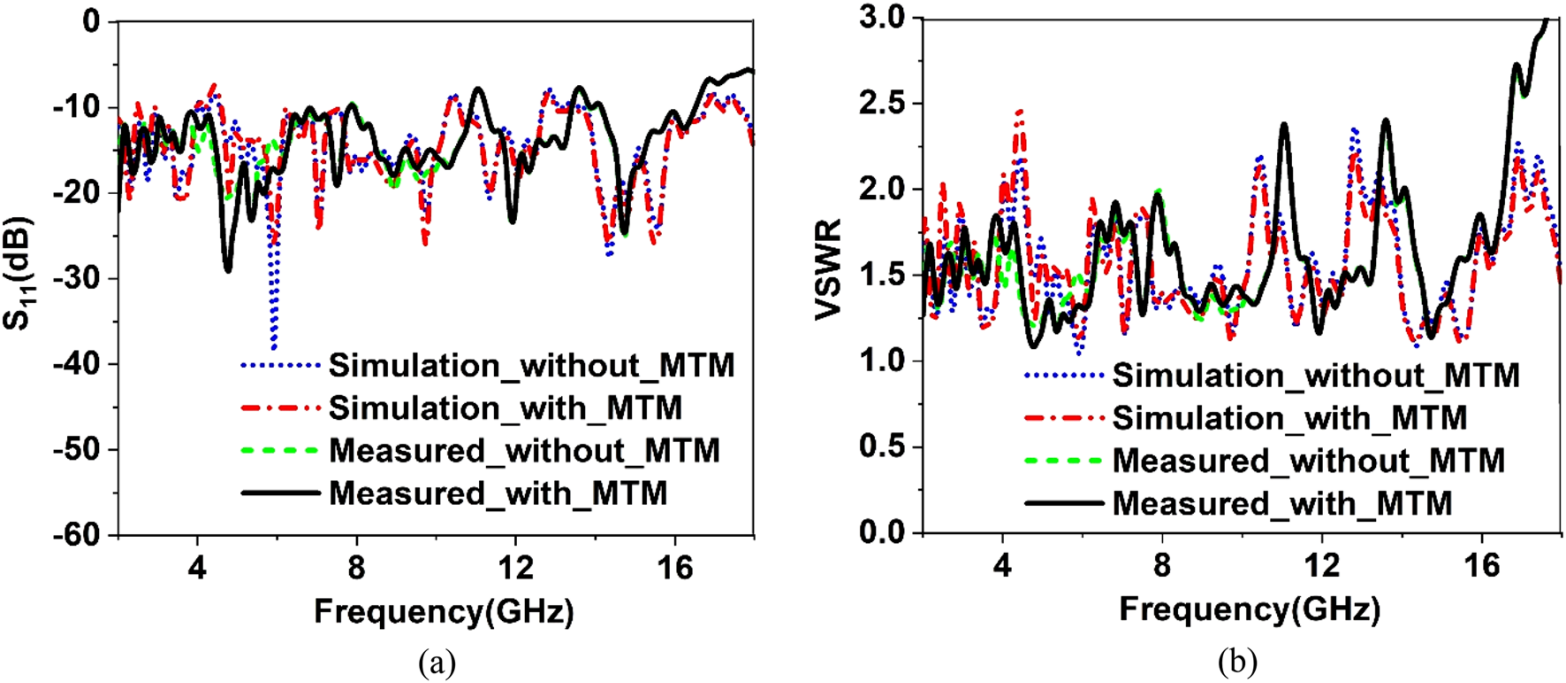
Comparison of simulated and measured results of the proposed antenna with and without metamaterial cover: (**a**) S_11_ (**b**) VSWR.

The validation of the gain enhancement of the TLPD antenna by using the MTM cover is confirmed through an experimental measure of the reflection coefficient(S_11_), voltage standing wave ratio(VSWR), efficiency, gain, and radiation pattern. Figure 35 shows the fabricated prototype of the fabricated antenna and an array of 21 × 14 unit cells of the proposed MTM. These two elements are placed at 30 mm away by using a small polystyrene block. In Fig. 36a, the proposed antenna system is connected to the vector network analyzer (VNA) to obtain the measured scattering parameter, whereas in Fig. 36b, nearfield measurement arrangement is shown by using Satimo nearfield measurement system. Simulated and measured S_11_ of the TLPD antenna with and without MTM cover is shown in Fig. 37a. A close observation of this Figure indicates that antenna with and without MTM exhibits nearly the same multi-band characteristics. However, a minor mismatch in bandwidths and resonance frequencies between measured and simulation results is noticed. Fabrication tolerances may be the one reason for this mismatching. In measurement, polystyrene block is used as a spacer between antenna and MTM array which also causes a change in bandwidth and resonance frequencies. Moreover, calibration error and losses in the cable are also added to the measured result that increases the mismatching between measured and simulation results.

**Figure 38:**
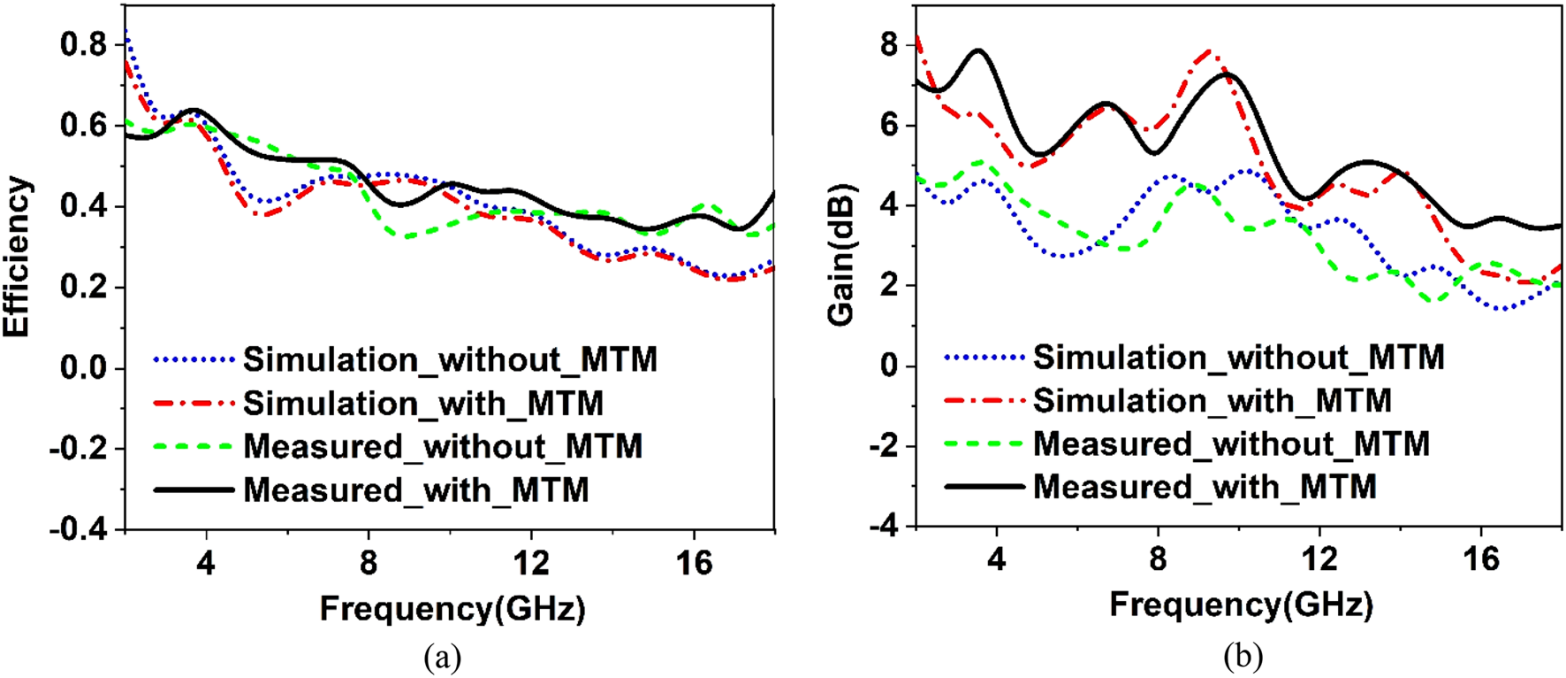
Comparison of simulated and measured results of the proposed antenna with and without metamaterial cover: (**a**) Efficiency (**b**) Gain.

**Figure 39:**
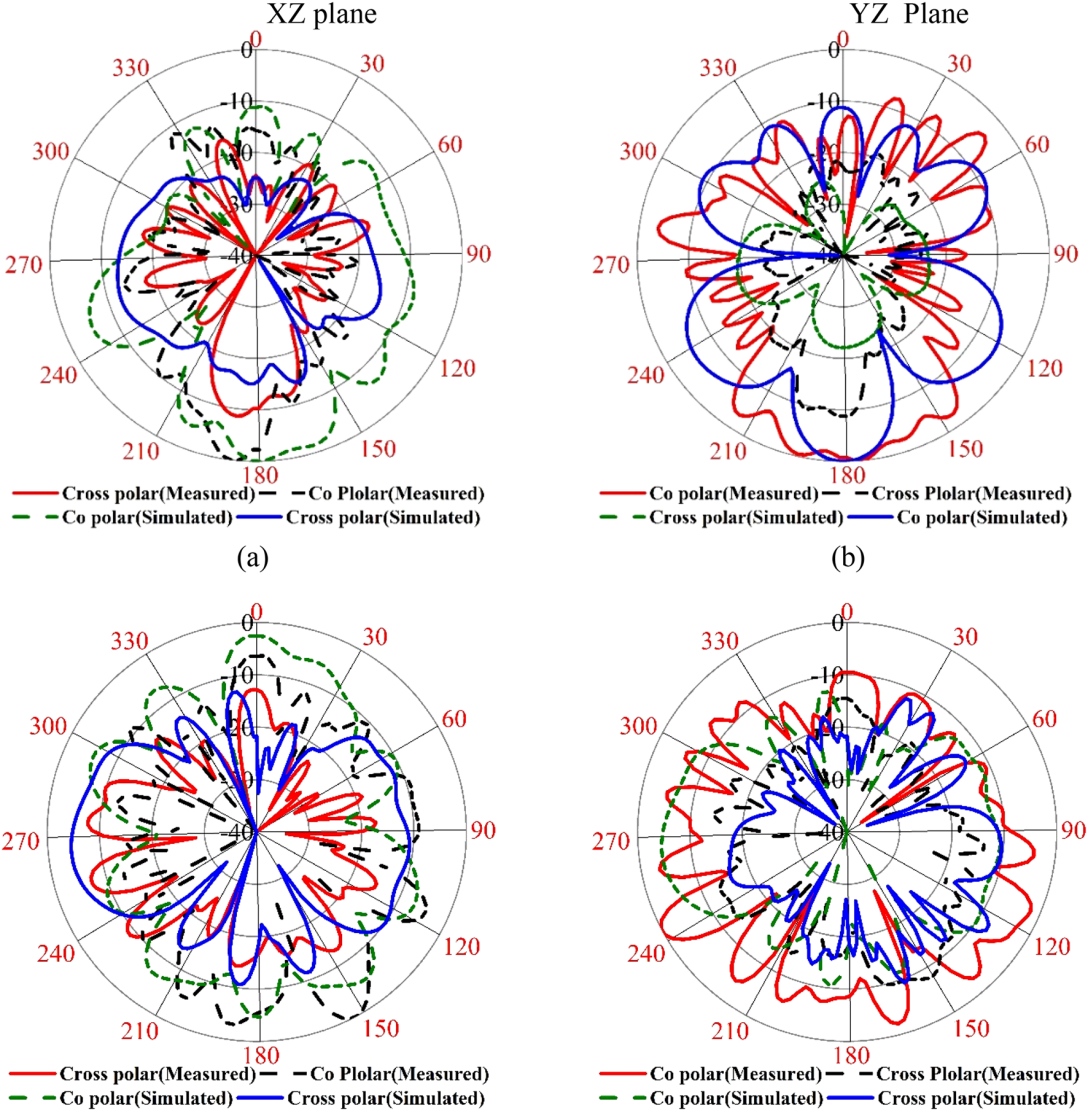
Simulated and measured radiation pattern of the proposed metamaterial with antenna (**a**) 5.5 GHz and (**b**) 11.70 GHz.

Voltage standing wave ratio (VSWR) is associated with the reflection loss and can be represented as $VSWR = \frac{{1 - \left| {S_{11} } \right|}}{{1 + \left| {S_{11} } \right|}}$. It is a very important parameter for various practical applications. A low value of the VSWR indicates that the antenna reflects less power. When the value of VSWR is less than 2, the antenna is considered well-matched, and additional impedance matching is not essential. On the other hand, if it is more than 2, more power is reflected, and accurate information transfer may also be hindered due to this large reflection. For this reason, the simulated and measured VSWR of the antenna with and without MTM is compared in Fig. 37b. A comparison of VSWR presented in Fig. 37b shows a close similarity between measured and simulated results. VSWR is less than two at most of the frequencies indicating good impedance matching. VSWR of higher than 2 is noticed at out bands near 11.5 GHz, 13.6 GHz, and beyond 16.5 GHz, meaning that antennas incur high energy loss in those frequency ranges. Around 1.5 VSWR is experienced in the frequency ranges of 2.2–2.5 GHz, 2.78–2.92 GHz, 3.48–3.6 GHz, 4.46- 6.2 GHz, 7.3 -7.6 GHz, 8.4- 10.6 GHz, 11.7–13.24 GHz, and 14.48–15.3 GHz, indicating that in these bands the reflected power is less than 4% with well impedance match. The antenna's efficiency is presented in Fig. 38a, whereas gain is represented in Fig. 38b. The antenna's efficiency is nearly 60% at the low frequency, but as the frequency increases, it gradually decreases. Since FR-4 is associated with the loss at high frequencies, efficiency gradually decreases as the frequency increases. On the other hand, the gain plot shows that metamaterial cover helps to increases the measured gain of the antenna, similar to the simulated gain associated with the antenna with MTM. At some frequency ranges, the measured gain is well above the simulated gain when metamaterial is used with the antenna. It is the effect of the polystyrene block that is used as the spacer within the antenna and MTM array. The simulated and measured normalized radiation patterns at 5.5 GHz and 11.70 GHz are depicted in Fig. 39. The co-polarized radiation patterns at 5.5 GHz are in very good agreement in simulation and measurement. It is seen from the radiation patterns that the antenna is more directive at lower frequencies at 5.5 GHz in the Z direction. The cross-polarization is low compared to co-polarization. Overall simulated and measured results exhibit some differences because of errors associated with the experimental study. Lack of proper impedance matching of SMA connector with antenna system and coaxial cable, soldering effect, noise associated with the long extended coaxial cable have some impact on the overall performance of the antenna.

## Conclusion

A quad-band metamaterial with six resonances covering S, C, X, and Ku-bands is presented in this article. The unit cell is formed on a low-cost FR-4 substrate with a small electrical dimension of 0.063λ × 0.063λ, where λ is calculated at the lowest resonance frequencies of 2.38 GHz. The resonances occur at 2.38, 4.24, 5.95, 9.55, 12.1, and 14.34 GHz, respectively, with negative permittivity, near-zero permeability, and near-zero refractive index in the vicinity of these resonances. The array performances are also investigated in simulation and experiments; those exhibit multiple resonances covering the target bands with little distortion. The calculated EMR shows a high value of 15.75 that expresses the compactness of the design. To understand the metamaterial properties, electric and magnetic fields along with the surface current are analyzed. The equivalent circuit of the unit cell is designed, and it is validated by simulation in ADS and cross-checked the output (S_21_) with the CST result. Both results provide a close similarity. The absorber property has been checked with the copper backplane of the unit cell, and it provides four major absorptions of 99.6%, 95.7%, 99.9%, 92.7% absorption peaks with Q-factor of 28.4, 34.4, 23, and 32 at 3.98, 5.5, 11.73 and 13.47 GHz, respectively indicating it’s application possibilities for sensing and detecting purposes. ENG with near-zero permeability and near-zero refractive index properties makes this MTM important for high gain antenna-based sensor applications offering about 73% gain enhancement when applied as superstrate of a TLPD antenna. Due to its compact size, high EMR, multi resonance behavior with ENG, near-zero permeability, and near-zero refractive index, the proposed MTM can be utilized for microwave applications, especially for gain enhancement of multiband antennas.
